# Emergence of a Distinct Picobirnavirus Genotype Circulating in Patients Hospitalized with Acute Respiratory Illness

**DOI:** 10.3390/v13122534

**Published:** 2021-12-17

**Authors:** Michael G. Berg, Kenn Forberg, Lester J. Perez, Ka-Cheung Luk, Todd V. Meyer, Gavin A. Cloherty

**Affiliations:** Infectious Diseases Research, Abbott Diagnostics, 100 Abbott Park Road, Abbott Park, IL 60064, USA; kenn.forberg@abbott.com (K.F.); lester.perez@abbott.com (L.J.P.); Ka-Cheung.Luk@abbott.com (K.-C.L.); todd.meyer@abbott.com (T.V.M.); gavin.cloherty@abbott.com (G.A.C.)

**Keywords:** picobirnavirus, virus discovery, next-generation sequencing, respiratory illness, metagenomics

## Abstract

Picobirnaviruses (PBV) are found in a wide range of hosts and typically associated with gastrointestinal infections in immunocompromised individuals. Here, a divergent PBV genome was assembled from a patient hospitalized for acute respiratory illness (ARI) in Colombia. The RdRp protein branched with sequences previously reported in patients with ARI from Cambodia and China. Sputa from hospitalized individuals (*n* = 130) were screened by RT-qPCR which enabled detection and subsequent metagenomic characterization of 25 additional PBV infections circulating in Colombia and the US. Phylogenetic analysis of RdRp highlighted the emergence of two dominant lineages linked to the index case and Asian strains, which together clustered as a distinct genotype. Bayesian inference further established capsid and RdRp sequences as both significantly associated with ARI. Various respiratory-tropic pathogens were detected in PBV+ patients, yet no specific bacteria was common among them and four individuals lacked co-infections, suggesting PBV may not be a prokaryotic virus nor exclusively opportunistic, respectively. Competing models for the origin and transmission of this PBV genotype are presented that attempt to reconcile vectoring by a bacterial host with human pathogenicity. A high prevalence in patients with ARI, an ability to reassort, and demonstrated global spread indicate PBV warrant greater public health concern.

## 1. Introduction

A majority of emerging infectious diseases are zoonoses, resulting from viruses in animal reservoirs that cross species barriers into humans. Globalization, international travel, changes in the environment and climate, and encroachment of humans into natural habitats, have all facilitated the global spread of outbreaks such as Ebola, Zika, HIV/AIDS, MERS, SARS, and Nipah. Respiratory viruses in particular, such as influenza and coronaviruses, in addition to morbidity and mortality, stoke worldwide fear and untold economic damage as their ease of transmission makes it difficult to control. As of this writing, SARS-CoV-2 has resulted in nearly 191 million cases and 4.1 million deaths (Johns Hopkins University, Baltimore, MD, USA, https://coronavirus.jhu.edu/ accessed on 22 July 2021), with new variants of concern emerging with each passing week [1,2,3]. SARS-CoV-2-specific molecular, serologic and rapid diagnostics, NGS surveillance, and vaccines have all been critical components of our public health response. To anticipate the next outbreak, metagenomic next-generation sequencing (mNGS) offers an unbiased approach to detect any known virus, bacteria, fungus, and parasite and identify novel, divergent pathogens that are otherwise undetectable with conventional molecular methods and require specific targeting or growth in culture [4,5]. Harnessing this technology has informed clinical diagnosis and aided in the discovery of a multitude of new viruses [6,7]. Acute respiratory illnesses encompass a wide spectrum of symptoms, ranging from rhinitis and phyrangitis to bronchitis and pneumonia, and as such, are caused by a litany of viruses (corona-, rhino-, adeno-, paramyxo-, herpes-, influenza, etc.), bacteria (*Streptococcus*, *Mycoplasma*, *Haemophilus*, *Klebsiella*, etc.), and fungi (*Candida*, *Histoplasma*, *Blastomyces*, etc.) [8]. It is imperative that surveillance of respiratory infections remains vigilant and that we proactively search for new viruses to be better prepared for the next pandemic.

Picobirnaviruses (PBV) are small (pico), two-segmented (bi), double-stranded RNA viruses primarily associated with gastroenteritis and diarrhea. Initially discovered in fecal samples from both humans and pigmy rats in Brazil, the virus is non-enveloped and contains two RNA segments that can differ in size depending on whether they are Genogroup I (2.3–2.6 kb and 1.5–1.9 kb) or Genogroup II (1.75 and 1.55 kb) strains [9,10]. Their highly heterogeneous sequences have been detected in a wide range of animal species worldwide, including everything from farm animals, reptiles, domestic pets, and wild birds, to non-human primates and untreated sewage [11]. The close relatedness of porcine and human strains and additional documented examples of interspecies transmission in chickens point to frequent crossover events and circulation between hosts [12,13,14]. Indeed, unlike other viruses that have co-evolved with their host, PBV strains do not segregate into distinct clades by species. Rather, the simple capsid appears to have acquired a generalized means of invading cells, easily transmitting from one host to another without restriction, although direct evidence of intracellular infection is still lacking [15,16]. Their high genetic diversity is further pronounced by the detection of multiple PBV strains within individuals [17]. Taken together, these observations have prompted the current debate around whether PBVs are even animal viruses. The presence of prokaryotic ribosomal binding sites (Shine Delgaro sequence) in 5′UTRs and alternative mitochondrial genetic code usage in some genera suggest these viruses may actually infect bacteria or fungi, resembling the segmented dsRNA *Cystoviridae* phages and *Mitoviridae* families, respectively. Thus, their detection at vertebrate mucosal sites may simply be a result of infection by these prokaryotic or unicellular eukaryotic hosts [18,19].

Nevertheless, studies indicate PBV can persist chronically, with periods of large shedding interspersed by periods of silence, and that some hosts can serve as asymptomatic reservoirs [20,21]. This implies the virus is adapted to the host and may underscore why pathogenicity (e.g., diarrhea) is often seen in those co-infected with other enteric viruses like rotavirus, calicivirus, and astrovirus [22,23]. Similarly, while PBVs have been found in humans as the ‘sole’ pathogen in cases of watery diarrhea and gastroenteritis, this is often in immunocompromised patients [24,25]. For these reasons, PBVs are currently viewed as opportunistic pathogens, with those who have underlying conditions or co-morbidities most at risk for infection. PBVs have also been detected in the respiratory secretions of pigs and humans [14,26]. Recently, novel PBV strains were detected in Uganda in two patients hospitalized with severe, acute respiratory illness [27]. In our search for novel pathogens causing respiratory illness, we surveyed patients hospitalized in South America and discovered a novel strain of PBV. We developed and applied an automated, high-throughput RT-qPCR assay which identified additional variants with high similarity to the index still in circulation, as well as strains previously reported in individuals from Cambodia and China with respiratory infections [28]. Our insights into their phylogenetic relatedness, prevalence, and association with disease warrant further examination of these picobirnavirus lineages.

## 2. Materials and Methods

### 2.1. Ethics Statement and Specimens

The initial 16 human specimens (sputum, ETA, and BAL) from MRN Diagnostics (Franklin, MA, USA) were collected in late 2016–early 2017 from four hospitals in Colombia, South America: Barranquilla, Cucuta, Medellin, and Valledupar. Patients were de-identified, provided oral informed consent, and the study (MRN GP 072014) received IRB approval. An additional 50 sputum specimens from MRN Diagnostics were collected in Colombia during June–July 2019. Left-over sputum from 50 individuals at a tertiary care hospital in New York City were collected in December 2018–January 2019 by New York Biologics (Southampton, NY, USA). Study number 01015-18 received IRB approval IRB00007807. Sputum samples (*n* = 30) from US patients hospitalized in Florida were sourced from Boca Biolistics, LLC who retains IRB records (Pompano Beach, FL, USA). Specimens were collected independently of one another, thus the inclusion criteria and symptoms displayed differed between cohorts.

### 2.2. Sputum Pre-Treatment

Forty-eight samples were processed at a time in a laminar flow biosafety cabinet in a BSL-3 facility. Sputum samples (0.5 mL) were resuspended 1:1 *v/v* in freshly prepared 2X pretreatment buffer: 2X Benzonase buffer (40 mM Tris-Cl pH 7.5, 20 mM NaCl, 4 mM MgCl2), 0.2% DTT [29], Ultrapure Benzonase (250 U/0.5 mL; Sigma), Turbo DNase (200 U/0.5 mL; Sigma), DNAse I (20 U/0.5 mL; Roche). Diluted sputum was vortexed, mechanically disrupted with disposable pestles (≥10 passes depending on viscosity), and incubated for 3 h at 37 °C, with vortexing repeated every 45 min. Samples were centrifuged at 10,000 rpm for 2 min to pellet insoluble debris and 800 µL was transferred to a fresh tube.

### 2.3. Extraction

Pre-treated sputum was extracted on an m2000sp using the TNA + Proteinase K protocol (Abbott Molecular, Des Plaines, IL, USA). Nucleic acid was eluted in 60 µL and frozen at −80 °C until use.

### 2.4. In Vitro Transcription

Positive controls for qPCR were subcloned into the pBlueScript plasmid, linearized with *HindIII*, and RNA was generated by in vitro transcription using the MEGAscript kit (Ambion, Austin, TX, USA).

### 2.5. PBV RT-qPCR Assay

A complete description of RT-qPCR assay volumes and cycling parameters, along with primer and probe sequences, are found in the online Appendix. Briefly, two separate reactions were performed in 96-well plates to detect PBV capsid or RdRp. In each case, 40 µl of mastermix containing AgPath-ID One-Step RT-PCR (Life Technologies) reagents, 1 mM MgCl2, primers (0.4 µM), and probe (0.3 µM) was combined with 10 µl of sample nucleic acid, with ROX added as the reference dye. Results were analyzed in MultiAnalyze software.

### 2.6. mNGS Library Prep and Sequencing

mNGS libraries were prepared as described [30]. Briefly, total nucleic acid from PBV+ sputum was converted to cDNA with random primers and Superscript III (SSRTIII) 1st Strand reagents (Life Technologies, Carlsbad, CA, USA), followed by 2nd strand synthesis with Sequenase V2.0 T7 DNA pol (Affymetrix, Santa Clara, CA, USA). Nextera XT was used to create barcoded, metagenomic NGS libraries which were multiplexed (*n* = 8) and sequenced on 3 MiSeq runs, with those requiring additional throughput re-sequenced on a HiSeq instrument.

### 2.7. mNGS Analysis

Metagenomic data were analyzed by SURPI and an Abbott-internal (DiVir) pipeline [31]. Briefly, SURPI uses the SNAP nucleotide aligner to remove human reads, compares remaining reads to all sequences in NCBI nt (2019 release), and then taxonomically classifies those with a match. Viral and unmatched reads are de novo assembled, translated in six reading frames, and aligned to a viral protein database using RAPSearch. Unmatched reads were probed further for divergent viral sequences using the psiBLAST algorithm in DiVir. Viral and bacterial reads identified by SURPI were normalized across experiments to determine fold change in abundance.

### 2.8. Pairwise Amino Acid Identity

An R/Shiny application was developed to align two amino acid sequences pairwise, measuring and plotting the rolling proportion of identical amino acids (AA) over a user-defined window, outputting mean and median identity values.

### 2.9. Phylogenetics

Phylogenetic analyses were performed on the entire coding regions of capsid and RdRp proteins. All available PBV sequences for both segments were downloaded from GenBank on 3 February 2021. After removing incomplete, poor quality and redundant sequences, a total of 477 capsid and 426 RdRp were included in the analyses. Alignment of amino acids with MAFFT E-INS-i and the refined taxonomic assignment of picobirnavirus sequences are described in Perez, L et al. [32]. All datasets were analyzed by the maximum likelihood (ML) phylogenetic approach computed with the IQ-TREE program [33]. IQ-TREE was also used to select the best-fit model and determine the confidence levels for the branches by the Shimodaira test and 100,000 bootstrap replicates [34]. Sequences from PBV species 2 were used as the outgroup for RdRp. See Supplementary Tables S1 and S2 for a listing of references used in trees.

### 2.10. Phylogeny-Trait Association Analysis

The association between clinical respiratory disease and the phylogeny of both PBV genomic segments was assessed using the Mr. Bayes BaTS software for the 477 capsid and 426 RdRp sequences by considering two states (e.g., ±clinical respiratory condition) [35]. Values of the association index (AI), parsimony score (PS) statistics, and the level of clustering in individual locations using the monophyletic clade (MC) size statistic were all calculated based on the posterior samples of trees produced by Mr. Bayes 3.2.7 using the BaTS program [36]. The null distribution for each statistic was estimated with 100,000 replicates of state randomization.

### 2.11. Statistics

Pearson’s chi-squared test was used to determine whether there is a dependency between infection with Mycobacterium tuberculosis and picobirnavirus.

### 2.12. NCBI Accessions

Partial and full-length sequences were deposited in GenBank under the following accessions: OL875303-OL875351. Raw data was deposited under BioProject number PRJNA789273 and SRA study SRP350910.

## 3. Results

### 3.1. Discovery of a Novel Picobirnavirus

**Figure 1:**
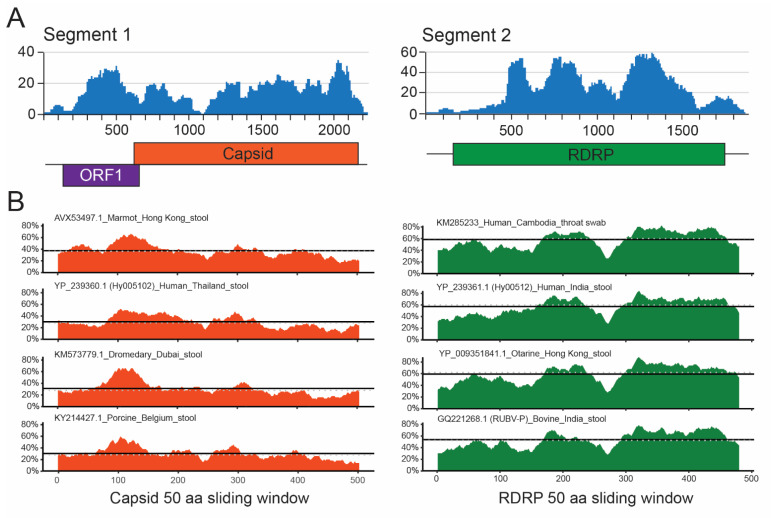
Discovery of a divergent PBV in a patient with acute respiratory illness. (**A**) Genome coverage plots of NGS data for segments 1 and 2. Segment 1 is approximately 2.5 kb long and encodes a hypothetical, hydrophilic protein (ORF1) of 168 aa and a 527 aa capsid protein in a separate reading frame. Segment 2 is approximately 1.7 kb long and encodes the 530 aa RdRp. (**B**) Pairwise amino acid alignment (50 aa sliding window) of the ABT3406 capsid (orange; left) and RdRp (green; right) with representative picobirnavirus strains. The mean (solid line) and median (dotted line) identities overall are approximately 35% and 60% for capsid and RdRp, respectively.

To discover novel human viruses involved in acute respiratory illness, specimens from patients hospitalized with severe symptoms were screened by mNGS. Sputum specimens (*n* = 16 patients) were collected from August 2016–February 2017 from hospitals in 4 different cities of Colombia, South America. Bacterial pathogens are known to cause respiratory or nosocomial infections, such as Klebsiella, Acinetobacter, and Stenotrophomonas were detected (Supplemental Table S3). Influenza A, Herpes simplex virus 1, known to cause respiratory infections in the immunocompromised, and Aichivirus A, a picornavirus typically causing gastroenteritis, were each found in different individuals. Sample 08853406, from a 29-year-old male hospitalized in Barranquilla, was enriched for Haemophilus parainfluenzae/influenzae reads, but also had two strong matches to porcine picobirnavirus and several other sequences with remote amino acid homology to picobirnaviruses. Additional sequences with homology to each PBV genome segment were identified by RAPsearch and psiBLAST. These reads served as seeds to de novo assemble the entire genome of 4119 nt from just 676 out of 2,327,238 total reads, at a coverage depth of 19X (Figure 1A).

For segment 1 (2251 nt), a 5′UTR of 144 nt with 66% AT-richness precedes ORF1 (nt 147–651), a 168 aa hypothetical protein with a predicted molecular weight of 18.7 kDa and acidic pI of 5.93. ORF1 possesses the ExxRxNxxxE repeat motifs of unknown function observed in other picobirnaviruses [37]. The top BLASTp hit for ORF1 is porcine PBV (YP_009241384.1) with only 33% identity and 47% positivity. ORF2 (nt 657–2238) encodes a 527 aa capsid protein with a predicted molecular weight of 57.8 kDa and a basic pI of 8.42. The top BLASTp hit is Grey Teal PBV (QD92430.1) at 44% identity and 63% positivity. Pairwise amino acid alignments with representative capsids from various species all derived from stool indicate an overall 35% identity to the novel sequence (Figure 1B; left) [11,16,38]. Segment 2 (1892 nt) encodes only the RdRp protein (nt 5–1594) of 530 aa, with a predicted molecular weight of 61.1 kDa and a neutral pI of 7.69. It is preceded by a 4 nt (incomplete) 5′UTR and followed by a 300 nt 3′UTR. Its top BLASTp hit is otarine (California sea lion) PBV (AMP18954.1) at 64% identity and 75% positivity. Pairwise amino acid alignments here indicate an average identity of 60%, in agreement with the greater degree of conservation among RdRp sequences (Figure 1B; right). The novel strain is referred to hereafter as ABT3406, and in keeping with currently established nomenclature, deposited in GenBank as follows: GI/PBV/human/Colombia/ABT3406/2016.

### 3.2. ABT3406 RdRp Branches with Respiratory PBV Strains

All available picobirnavirus sequences in GenBank were retrieved for phylogenetic analysis at the time of discovery (December 2018) and prior to submission (February 2021). Full-length capsid sequences (*n* = 423) were aligned by amino acid and classified into 3 species [32]. Long branch lengths and the absence of virus–host co-evolution patterns reflect the heterogeneity of strains and a high number of accumulated mutations indicative of adaptation to new hosts and the apparent interchangeability of PBV capsids [16]. Consistent with the BLAST results, ABT3406 branched with MK204396 from Grey Teal ducks (*Anas gracilis*) [38] (Supplemental Figure S1).

**Figure 2:**
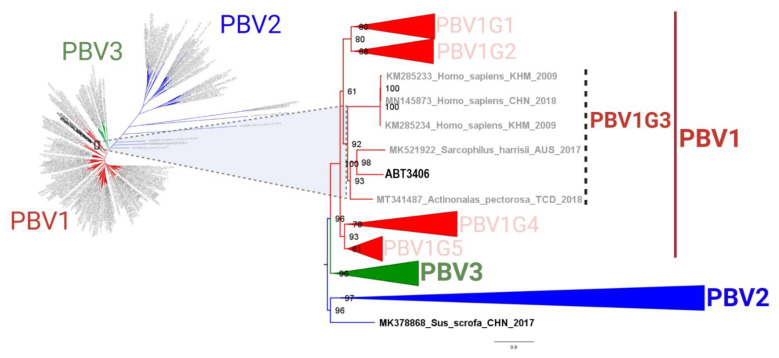
RdRp of ABT3406 branches with other respiratory PBV strains (left panel). Phylogenetic tree based on the complete deduced amino acid sequences of PBV RdRp from the index case (ABT3406) and 403 non-redundant genomes available in GenBank. The maximum likelihood (ML) method with an LG+F+R10 model was used and the main lineages for the three PBV species are denoted by colors (PBVR1: red, PBVR2: blue, and PBVR3: green), and collapsed (except PBVR3) for visualization purposes (right panel). Phylogenetic relationship between ABT3406 and other PBV viral strains linked to respiratory disease in humans all cluster together into genotype 3 of PBVR1.

Phylogenetic analysis of RdRp sequences has historically involved interrogation of a 165–200 nt/55–66 aa region spanning amino acids 209–264 [39]. Full-length sequences, however, yield more reproducible trees which classify picobirnaviruses into three species, rather than two genogroups [16,32,39] (Figure 2). To readily visualize ABT3406, species PBVR2 and PBVR3 and all genotypes within PBVR1 were collapsed. In a tree containing 403 full-length sequences, ABT3406 branched with MK521922 (Tasmanian devil; 57.2% AA identity | 70.8% AA similarity) and MT341487 (freshwater mussel; 50.8% | 62.5%), along with three notable strains: KM285233 and KM285234 (human; 57.2% | 72.0%), both obtained in 2009 from upper respiratory swabs of two patients in Cambodia (GenBank: Mishra, N. and Lipkin, W.I.), and MN145873 (human; 56.6% | 71.7%), from a pediatric respiratory infection patient in China (Figure 2) [28]. Thus, while the overwhelming majority of PBV RdRp sequences in GenBank are derived from stool samples, ABT3406 branched with a small handful of sequences implicated in respiratory illness.

### 3.3. Quantitative PCR Screen Reveals High Prevalence of PBV among Hospitalized

**Figure 3:**
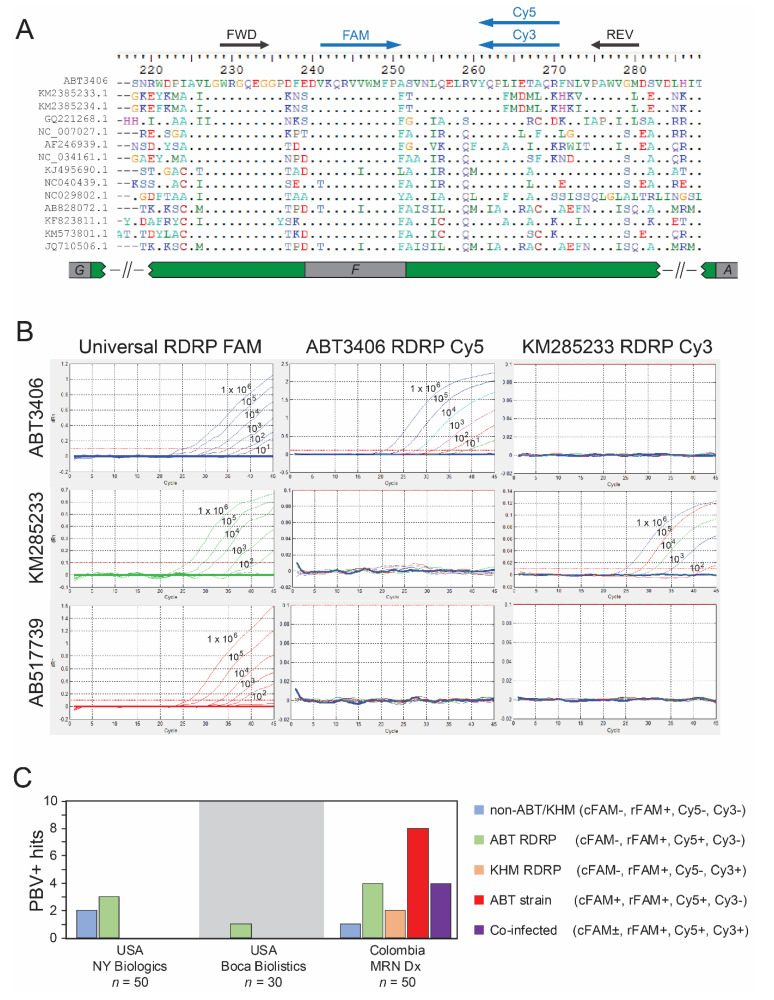
Quantitative PCR screen identifies PBV-positive patients hospitalized for acute respiratory illness. (**A**) Amino acid alignment of representative PBV from species PBVR1 for the qPCR target region in RdRp (polymerase motif F) and position of primers (FWD, REV) and probes (FAM, CY3, CY5) within the amplicon. (**B**) Amplification curves of serially diluted IVT positive controls (10^1^–10^6^ copies/mL) for ABT3406, KM285233, and AB517739 (rows 1–3) and their detection by FAM, CY5, and CY3-labeled probes (columns 1–3). The FAM probe binds to sequences conserved across PBV while CY5 and CY3 probes discriminate strains. Detection of ABT3406 capsid IVTs is shown in Supplemental Figure S2. (**C**) Results of RT-qPCR screen of 130 patients hospitalized with acute respiratory symptoms. Sputum was sourced from NY Biologics (NY, USA; *n* = 50), Boca Biolistics (Florida, FL, USA; *n* = 30) and MRN Diagnostics (Colombia, South America; *n* = 50). PBV+ RT-qPCR probe reactivity profiles are indicated in the legend and histograms are colored accordingly.

To understand the prevalence, identify additional strains, and assess genetic diversity, a quantitative PCR assay was designed to enable the detection of all picobirnaviruses and discriminate ABT3406 from other strains. An alignment of ABT3406 with reference sequences from human, chicken, pig, otarine, turkey, bovine, fox, camel, and monkey depicts the conserved region targeted in RdRp (Figure 3A). Redundant, degenerate primers generate an amplicon whose 5′ end is recognized by a universal, FAM-labeled probe (rFAM) situated in polymerase motif F. CY5- and CY3-labeled probes are unique to ABT3406 and KM285233, respectively, and recognize overlapping, mutually exclusive sequences at the amplicon 3′ end (Figure 3A). In a separate reaction, different primers and a FAM-labeled probe (cFAM) specifically target the ABT3406 capsid (Supplemental Figure S2). Therefore, the RT-qPCR assay provides four measurements detecting both PBV genomic segments, functioning as a discovery and diagnostic tool.

Serial dilution of in vitro transcripts (IVT) demonstrates dose dependency, sensitivities with LODs between 10–100 copies/mL, and the expected results for three classes of strains represented by ABT3406, KM285233, and AB517739 (Figure 3B). All were detected by the universal rFAM probe (column 1). For AB517739, illustrative of any PBV, this is the only reactive probe. The ABT3406-derived IVT is also detected by the RdRp CY5 (column 2) probe, while KM285233 is dually reactive with the RdRp CY3 (column 3) probe. For the cFAM capsid probe, only the ABT3406 strain is detected (Supplemental Figure S2).

Sputum samples (*n* = 130) were obtained from patients hospitalized with ARI. Specimens were collected in October 2018–July 2019 from two new locations in the United States (New York, NY, USA, *n* = 50; Florida, FL, USA, *n* = 30) and from the original medical facilities in Colombia (*n* = 50). Nucleic acid was tested for PBV by both capsid and RdRp RT-qPCR assays as well as with an Aichivirus A qPCR (Supplemental Figure S3). A total of 25 patients (19.2%) were positive for PBV with at least one probe (Figure 3C and Table 1); there were no Aichivirus A positives (0/130; data not shown). We identified five hits from New York, NY, USA: 2 rFAM+ (blue) and 3 rFAM+/CY5+ (green). Reactivity with only the universal RdRp probe indicates these may be altogether new PBV strains (‘any PBV’: non-ABT/non-KHM). For the latter three, this profile predicts an RdRp similar to ABT3406 combined with a different capsid. A single rFAM+ only (blue) sample was found in Florida, FL, USA. The remaining 19 hits all originated from Colombia. Four were rFAM+/CY5+ (green) and one was rFAM+ (blue). Eight resembled the ABT3406 index (cFAM+/rFAM+/CY5+; red) in having highly similar capsid and RdRp sequences, and two resembled the KM285233 strain from Cambodia (cFAM-/rFAM+/CY3+; orange). Finally, there appeared to be four dual ABT3406/KM285233 infections (cFAM±/rFAM+/CY5+/CY3+; purple). Estimated viral loads ranged considerably (log2–log6 copies/mL), but importantly probe Cts were of similar magnitude within a multi-reactive sample (e.g., Ct of rFAM ≈ Ct of CY5; Table 1).

### 3.4. Emergence of a Distinct PBV Genotype

**Figure 4:**
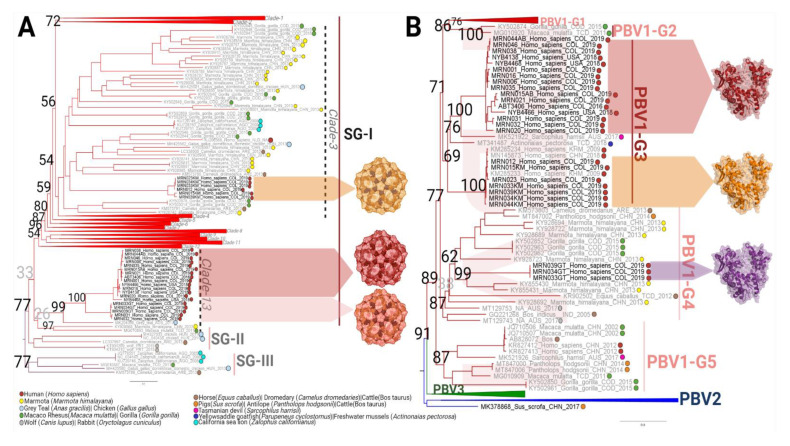
Respiratory tropism is defined by the RdRp protein (**A**) Phylogenetic tree of PBV capsids obtained in the current study (taxa denoted in black) and 422 non-redundant genomes available in GenBank. The complete deduced amino acid sequences were analyzed using the maximum likelihood (ML) method with an LG+F+R10 model. The main lineages and clades in which new sequences cluster are denoted. (**B**) Phylogenetic tree of PBV RdRp obtained in the current study (taxa denoted in black) and 403 non-redundant genomes available in GenBank. The complete deduced amino acid sequences were analyzed using the maximum likelihood (ML) method with an LG+F+R10 model. Due to the high number of taxa, genotype 1 of PBVR1 was collapsed for visualization purposes, as were unrelated PBVR2 and PBVR3 species. The hosts from which the different sequences have been identified are denoted by colored circles as shown in the legend.

Total nucleic acid (*n* = 25 hits) was converted into mNGS libraries for sequencing. The number of PBV reads identified by our analysis pipelines correlated inversely with qPCR values, and therefore those (*n* = 8) with Cts > 35 resulted in little to no coverage. Full and partial segments 1 and 2 sequences were recovered from 17 patients. Several had dual PBV infections, yielding a total of 25 capsid and 26 RdRp sequences. To avoid bias, consensus sequences were independently determined by mapping to a reference (e.g., ABT3406 or KM285233) and by de novo assembly, then merged into alignments by codon and trees analyzed by the maximum likelihood method (Table 1, Figure 4).

For capsid, we observed a clustering of nine sequences which include the ABT3406 index case: all are from Colombia (038, 044, 046, 006, 035, 015, 021, 001), have the cFAM+/rFAM+/CY5+ (red, purple) qPCR profile, and share >94% amino acid identity. Basal to this node is a mixture of five sequences from Colombia and New York. At 78–92% amino acid identity to ABT3406, these were all negative by capsid qPCR, but had similar overall profiles: cFAM-/rFAM+/CY5+ (green: 016, 4468, 4138) and cFAM-/rFAM+/CY5- (blue: -020, 4466). A separate branch indicating an earlier speciation event (not supported by bootstrapping) is populated with five sequences (-033, -034, -039, -031, -032), with only ~45% amino acid identity to the index capsid and all except -039 (weakly positive) were negative for capsid qPCR. All these sequences residing in clade 13 share a common ancestor found in Grey Teal ducks, MK204396 [38] (Figure 4A). By contrast, the capsids from cFAM±/rFAM+/CY3+/CY5± (orange) strains (-039, -034, -033, -012, -015, -023), linked to the ‘Cambodian’ RdRp, cluster together on a distant branch of Clade 3 and possess only 19% amino acid identity to ABT3406. Capsid sequences were never deposited for Cambodian (KM285233, KM285234) and Chinese (MN145873) strains. It is remarkable given the extreme heterogeneity of PBV, that capsids assembled for all individuals, including those co-infected (e.g., PBV-19-015, 033, 034, 039), branched only in these clusters and nowhere else on the tree.

The RdRp tree yielded a highly correspondent phylogeny, indicating genome segments within a patient are linked and constitute related, but distinct strains (Figure 4B). Despite the variability in qPCR profiles (cFAM±/rFAM+/CY5±), US and (4468, 4138, 4466) and Colombian (038, 044, 046, 006, 035, 015, 021, 001, 016, 020) RdRp sequences (*n* = 16) branched with the index case. These variants share 86–95% amino acid identity and demonstrate the strain’s expansion throughout the Western Hemisphere. RdRp proteins with the cFAM-/rFAM+/CY3+ profile (*n* = 7; -039, -034, -033, -044, -012, -015, -023) were also on the same branch. It was conceivable given the Cy3 probe reactivity that these would be related to the Cambodian/Chinese respiratory strains in Figure 2, however, we would not have predicted >96% identity to these references. This confirms the strain remains in circulation and has traveled from Asia to South America. These RdRps are only ~57% identical to ABT3406 and were CY5- (except dually infected 015), yet the lineages consistently branch together as genotype-3 of PBV species 1 with a bootstrap value of 69. The outliers in this tree are PBV-19-039, -034, -033 sequences within genotype 4, which BLAST with 52% identity to stool-derived sequences from different species, and ancestral to an Australian rabbit sequence. Their RdRps share only ~45% identity to the index, yet intriguingly all three were weakly positive for CY5 and linked to the ‘maroon’ cluster of capsids that speciated from the index (Figure 4A). Notably, all three of these individuals were co-infected with the ‘Cambodian/CY3+’ virus. PBV-19-031 and -032 provide a final evolutionary twist. Possessing an ABT3406-like RdRp and a maroon capsid, these individuals may represent a ‘missing link’. It is well documented and demonstrated here that multiple PBV strains can be found simultaneously in a host [40]. Due to the abundance of mono-infections identified, in which the pairing of genome segments was unequivocal, and taking into account equivalent NGS reads, we could conclude with reasonable certainty in dual-infection settings which capsid paired with which RdRp (Table 1, Figure 4).

### 3.5. RdRp and Capsid Are Both Associated with Respiratory Disease

To determine whether these distinct lineages (e.g., genotype, clade) were linked to the clinical outcome (phenotype: respiratory disease trait), we applied Bayesian inference (BaTS) analysis [35]. RdRp sequences from sputum form a monophyletic genotype (III) for which no stool-derived strains were included. Patients with PBV-19-039GT, -034GT, -033GT sequences in genotype 4 all have co-infections within genotype 3 and were omitted. Index ratios (IR) of observed versus expected values are an indication of the strength of the association, wherein 0 predicts a complete subdivision of the population and values approaching 1 suggest random mixing (panmixia). The null hypothesis (e.g., no greater trait association with adjacent taxa than due to chance) for phenotypic structure was rejected. Rather, an association index (AI) of 0.14 suggests the emergence of RdRp genotype 3 in species PBV-1 is linked to this clinical condition and the monophyletic clade (MC) statistics (4.20 observed vs. 1.05 expected) prove that the population is phylogenetically divided by these two states (Supplemental Table S4). A parsimony score (PS) of 0.22 eliminates the randomization of this genetic trait as an explanation. All the parameters (AI, PS, MC) were in agreement, indicating this particular clade contains the genetic signature linking it to respiratory disease.

BaTS analysis was similarly applied to capsid sequences. Intuitively, with the ‘Abbott’ and ‘Cambodian’ clades separated on the tree, one might have expected the association with respiratory disease to be weaker (Figure 4A). However, the results were even more convincing than for RdRp, with the IR at 1.9 × 10^−4^, PS at 0.08, and the MC (CRC) at 19.0 observed versus 1.38 expected (Supplemental Table S4). To eliminate ambiguity in terms of which strains were associated with respiratory illness, removing dually infected individuals from the Cambodian clade only served to improve the BaTS statistics. While we cannot account for representation biases in GenBank and the possibility that GI-derived sequences may have also caused ARI symptoms in these same strains, based on available annotations the RdRp and capsid sequences identified here were both strongly associated with respiratory illness.

### 3.6. PBV Is Typically Present as a Co-Infection

**Figure 5:**
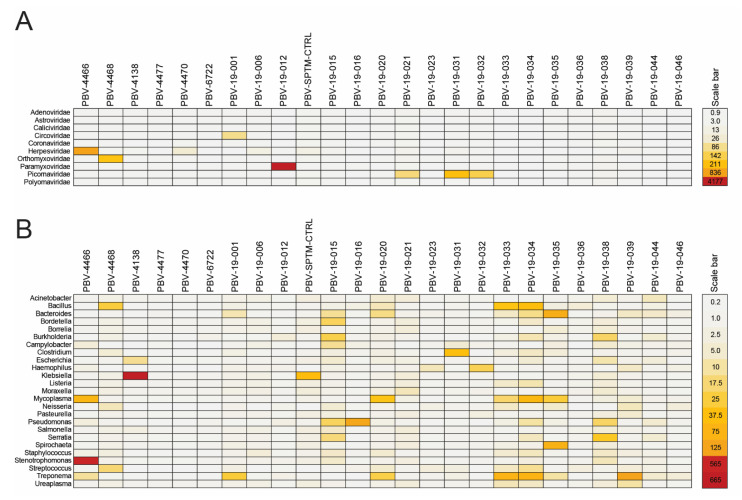
Co-infections are found in majority of PBV+ individuals. (**A**) Viral and (**B**) bacterial reads identified by SURPI from agents with known respiratory and gastrointestinal pathogenicity were sorted by family and genus, respectively. Read counts were normalized within samples, expressed as reads per million (RPM), and compared to other samples by calculating fold change relative to the median RPM number. Scale bars depict the magnitude fold change for each heat map. A 100 (absolute) read cutoff was established for bacteria, resulting in no comparisons for *Legionella* spp., *Chlamydia pneumonia*, and *Mycobacterium tuberculosis*.

Metagenomics permitted addressing whether picobirnaviruses represent an opportunistic infection that may exacerbate disease and are always secondary to a primary viral, bacterial, or fungal respiratory infection, or if they are the sole pathogen present and the presumed cause of illness. HHV-1, EBV, rhinovirus-A, respirovirus-3, and influenza-A reads were individually detected in samples ranging from 41–1712 reads per million, while in others with betacoronavirus, enterovirus-D, and HHV-2 there were fewer total reads (<100) (Table 2, Figure 5A). Likewise, several bacterial genera, including *Streptococcus*, *Haemophilus*, *Stenotrophomonas*, and *Klebsiella* were enriched (Table 2, Figure 5B). Often times, phages specific to these bacteria (e.g., *Klebsiella* and *Pseudomonas* in PBV-4138 and PBV-19-016, respectively) were also detected in abundance to confirm this infection was present. Heat maps illustrate the fold increase in viral and bacterial reads relative to the other PBV+ sputum samples sequenced (Figure 5). No fungal infections of significance were observed. While *Mycobacterium tuberculosis* reads were only sparingly detected by mNGS, 7/18 (39%) PBV+ patients from Colombia also had a diagnosis of pulmonary TB compared to 31/50 (62%) overall. Chi-square analysis indicated a patient was twice as likely to have either TB or PBV alone than to be co-infected (*p* = 0.011). For all 130 patients screened, there was no discernable difference in median age for PBV+ (60.5 years) versus PBV- (56.5 years) (Table 2). Thus, while co-infections were common in patients, the data illustrate that these picobirnavirus strains were not linked to one or more specific respiratory bacteria or fungi.

As with gastroenteritis, with 21/25 PBV+ samples co-infected, PBV appears to also be an opportunistic infection of the respiratory tract, although the order of onset for each is unknown (Figure 5). However, four patients did not show enrichment for other microbes, two of which had PBV viral loads ≥104 cp/mL, arguing it may be the sole pathogen causing symptoms or providing the initial insult (Table 2). Interestingly, these two individuals have clear sputum coloration and chills, consistent with a viral, acute upper respiratory infection, while the two with lower viral loads have yellowish tinges perhaps indicative of a resolving infection (Table 2). Metagenomics has the added advantage of being able to discriminate samples and rule out cross-contamination. The RdRp and capsid consensus sequences from 19-038, 19-044, and 19-046 are virtually identical, but clearly originate from different individuals: Cts, % PBV reads, bacterial profiles, and the fact that only 19-044 was co-infected with the KM285233-like strain, all indicate they are unique.

## 4. Discussion

A novel picobirnavirus strain was recovered from the sputum of a patient hospitalized in Colombia for acute respiratory illness. Picobirnaviruses infect a myriad of hosts, their sequences are highly variable, and they can be found in people with or without disease, thus it was initially difficult to ascertain the significance of this discovery. However, recent reports have taken note of their presence in respiratory illnesses, some of them severe, and others now appreciate PBV infects airways of animal reservoirs [26,27,41]. From the hundreds of PBV sequences deposited in GenBank, the ABT3406 RdRp branched with the infinitesimally small number of strains recovered from humans with respiratory ailments. It was conceivable that few if any additional ‘hits’ might be found with our screening efforts, and that these would likely represent sporadic cases with completely unrelated sequences. On the contrary, we found a high prevalence of PBV (19.2%), the majority from Colombia, and all were either related to the index case or the isolates from Cambodia and China previously implicated in respiratory illness. Extraordinary genetic diversity and multi-PBV infections are typically the norm. Sequences are either interspersed among a variety of human, vertebrate, and wastewater strains, despite having similar symptoms like diarrhea and GVHD, or it is the other extreme, where they are nearly identical and indicative of an outbreak [42,43]. Here, we observed PBV with phylogenetically related RdRps evolved over time and circulating over vast distances to emerge as a distinct genotype associated with ARI.

The robust RdRp RT-qPCR assay detected highly divergent strains over a broad range of titers and was used to screen in an automated and quantitative fashion which should now replace the limited efficacy and tediousness of manual RT-PCR and running of gels [39]. Indeed, qPCR led us to capsid and RdRp sequences with as little as 39% and 59% nucleotide identity to the index case, respectively. The amplicon region chosen appeared broad enough for the ‘universal’ probe to detect highly disparate sequences but specific enough to discriminate strains and identify dual infections. From a data integrity standpoint, viral loads varied considerably, with most samples positive in ≥2 channels, allaying concerns of contamination or false positives, respectively. Importantly, mNGS confirmed full-length sequences agreed with qPCR profiles. While false negatives were certainly a possibility, mNGS of PBV qPCR negatives (*n* = 105) on a HiSeq (>15 million reads/library) confirmed the absence of PBV in these sputum samples (data not shown).

**Figure 6:**
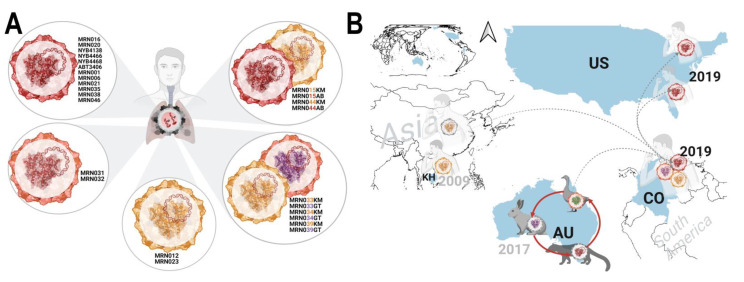
Respiratory PBV strains circulating in the Western Hemisphere originated from Asia and animal reservoirs. (**A**) Patient IDs are listed adjacent to capsid/RdRp combinations (single or dual infections) following the same color scheme in Figure 4. (**B**) PBV strains from Asia have traveled to South America and onward to North America. The ABT3406 index case and related strains may have arisen from an enzootic cycle in Australia between grey teal ducks, Tasmanian devils, and rabbits.

Most studies on PBV rely upon a narrow region of RdRp to classify strains and fail to report the sequence of the highly divergent capsid [44]. PBV’s ability to reassort and the seemingly interchangeable nature of capsid to permit rapid adaptation to new hosts belies the demonstrated dependence of RdRp molecules bound to genomic RNA to interact specifically with the capsid during packaging [15,45]. It has been proposed that polymerase activity requires the presence of a capsid to ensure that dsRNA genomes are enclosed within a capsid and sequestered to prevent elicitation of an antiviral response [46]. Here we demonstrated which capsid and RdRp segments were consistently linked. For example, despite negative reactivity for cFAM and/or CY5, any sample with an RdRp sequence bearing resemblance to the index was found to be paired with a corresponding ABT3406 capsid of roughly similar identity (≥91%) and vice versa. This same RdRp–capsid linkage was true of CY3 positive strains having a high identity to Cambodian/Chinese strains. Five patterns were observed that consist of mono- and co-infections of these strains, as well as those with highly related capsids (-031, -032 and -033GT, -034GT, -039GT) (Figure 6A). RdRps clustered within a distinct genotype whereas ‘Abbott’ and ‘Cambodian’ capsids, sharing only 20% amino acid identity, were in separate clades. (Figure 4A). Regardless, Bayesian inference analysis established both segments were linked to the respiratory disease trait. In other segmented dsRNA viruses such as reoviruses, tropism can be determined by cell-selective replication efficiency, a process regulated by the viral RNA-dependent RNA polymerase protein (λ3) at a late, post-entry point in the viral life cycle following primary transcription and translation [47]. Unfortunately, porcine and human respiratory sequences collected in the Netherlands from Smits, et al. and nosocomial infections associated with severe ARI in Wakiso, Uganda from Cummings, et al. were not deposited in GenBank and could not be compared [26,27]. The latter were reported to resemble swine (KX374477.1) and camel (KM573801.1) strains, neither of which branched closely to those we identified [27]. Nevertheless, we anticipate the emergence of other phylogenetically unrelated PBV lineages implicated in respiratory illness.

Numerous examples exist of viruses transmitted by the fecal-oral route that infect the mucosal surfaces of both the alimentary and respiratory tracts, including adenoviruses, picornaviruses, and orthomyxoviruses. Our work confirms and extends recent observations that PBV infections are not restricted to the GI tract nor only involved in gastroenteritis and diarrhea [26,27,48]. Indeed, Smits et al. showed that the RdRp of respiratory PBV from pigs in Hong Kong likely descended from a related strain found in US wastewater [14]. Woo, et al. further demonstrated in cows, poultry, and monkeys that viruses from stool and throat swabs of an individual were one and the same sequence [41]. In contrast to our study, their heterogeneous sequences were widely distributed across the phylogenetic tree and not associated with animal or human disease. As with the respiratory PBV from Uganda and the Netherlands, these too were unrelated to our sequences. Regarding an association with ARI, a comparison to healthy individuals is lacking and there is no guarantee sequences annotated in GenBank were restricted to a particular anatomical site or sample type. However, in the latter case, it is reasonable to assume that an individual suffering from diarrhea would not be asked to provide sputum, just as someone on a ventilator would not provide a stool specimen. Persistent shedding over months in immunocompromised individuals (e.g., HIV+) has advanced the prevailing notion that PBV is an opportunistic infection that otherwise healthy, asymptomatic carriers are invulnerable to [11,25]. Indeed, following hematopoietic stem cell transplants and immunosuppression, the sudden appearance of PBV replication was predictive of graft-versus-host disease onset in 40% of cases [42,49]. In Legoff, et al., it was theorized that infection with another pathogen creates an inflammatory milieu or leads to an imbalance in the mucosal microbiome that leads to a burst in PBV replication [42]. Here, we did not have longitudinal samples to assess causality, but a majority of samples showed evidence of viral or bacterial co-infections or had been previously diagnosed with tuberculosis, suggesting the respiratory PBV strains we detected also represent opportunistic infections.

Still, while the inherent pathogenicity of PBV remains inconclusive, questions remain as to whether PBV is a mammalian virus at all, or simply a prokaryotic virus infecting resident flora of the gut microbiome [18,19,50]. Their ability to auto-proteolyze their capsid and invade liposomes suggests they are vertebrate viruses, unlike the related partitiviruses that infect unicellular organisms and fungi [15,51]. Unfortunately, the inability to culture the virus in mammalian cells has hampered demonstrations of Koch’s postulates and laying this controversy to rest, although propagating PBV in bacteria has failed as well [50]. PBV-like viruses found in animals (e.g., bats, crustaceans) using alternative translation codons typically cluster distantly from other PBV in Genogroup III/species R3), yet there are instances (e.g., mongoose, bat) where branching among species Genogroup I/species R1 is observed [52,53]. Our sequences were phylogenetically unrelated to all of these and produced intact ORFs for RdRp using the standard genetic code. However, as with all PBV, segments 1 and 2 here possessed the Shine Delgarno ribosome binding site (AGGAGG) upstream of the ATG start codon [18,19]. Thus, one model to explain our data is that respiratory-tropic bacteria-harboring PBV actually caused disease, and PBV as neutral bystanders were guilty by association.

Our metagenomic results challenge this notion in several respects, though (Figure 5). First, we did not observe fungal co-infections in any PBV+ individuals. Second, there was either a variety of different respiratory bacteria present or none at all, rather than one or more specific prokaryotic or unicellular eukaryotic hosts linked to PBV positives. Third, there were cases of virus-only co-infections or those with no other pathogen detected, indicating PBV does not require a bacteria or fungi to sustain or initiate an infection. Certainly, more conclusive evidence is demanded, but this data leaves open the interpretation that PBV are pathogenic animal viruses, either as the initial insult or as an opportunistic infection. Evidently, the CY3+ (KM285233) strain remains in circulation and has traveled from Asia to South America (Figure 6B). Given the phylogenetic relationships of CY5+ RdRp and cFAM+ capsid sequences to those recovered from the Tasmanian devil and Grey Teal ducks, respectively, we speculate these hosts interacted in Australia giving rise to a zoonosis from which ABT3406 descended, making its way eastward to Colombia and throughout the Americas (Figure 6B). Just how strains may have jumped from one host to another will require co-evolutionary analysis, however, the non-enveloped, environmentally resistant nature of PBV will have undoubtedly facilitated its spread in the absence of a direct transmission event. As with animal reservoirs for influenza, the virus can be excreted in their stool and through reassortment can change tropism to trigger emergence in an incidental (human) host. Thus, these competing models are in fact not mutually exclusive: whether as the primary or as an opportunistic infection, PBV can induce ARI symptoms while at the same time be transmitted via a prokaryotic host vector. Regardless, the key takeaway is that genotype III strains described here are uniquely associated with respiratory illness.

New classifications of respiratory viruses are being discovered such as bocaviruses (Parvo-) and redondoviruses (CRESS DNA), while emergent species (e.g., MERS, SARS-CoV-2) from established families (e.g., coronaviruses) have literally changed our way of life [54,55]. The COVID-19 pandemic has laid bare the need to be proactively searching for new viruses and prepared with diagnostics, therapeutics, and vaccines. Metagenomics has once again shown that picobirnaviruses are also involved in ARI [27,28]. To our knowledge, ours is the first study to leverage mNGS to link and fully sequence both capsid and RdRp, as well as define the presence of other bacterial and viral agents in Picobirnavirus infections. Many questions remain unanswered: Is it a human or a prokaryotic virus? Is the virus seasonal? Are only the immunocompromised affected? Does PBV cause respiratory symptoms or is it simply a bystander? What domains of RdRp determine respiratory tropism? The high prevalence observed, coupled with its ability to rapidly evolve, reassort its segmented genome, and crossover to other species, indicates a need for greater public health awareness and future studies of picobirnaviruses [13,56].

## 5. Conclusions

We applied next generation sequencing for virus discovery and determined that two phylogenetically related lineages of PBV circulating in different hemispheres were present in patients with ARI, and that both capsid and RdRp segments are linked to respiratory disease. Typically thought of as opportunistic gastrointestinal infections in the immunocompromised and now more recently considered prokaryotic viruses, this report challenges the prevailing understanding and should serve as a springboard for others to explore PBVs role in respiratory infections and their legitimacy as a human pathogen.

## Figures and Tables

**Table 1:** Results of secondary screen for PBV positives: qPCR and NGS mapping.

			Capsid-FAM	RdRp-FAM	RdRp-CY3	RdRp-CY5	PBV Segment 1			PBV Capsid Only		PBV Segment 2-RdRp			NGS Reads
Sample ID	Country	Year	Ct	Ct	Ct	Ct	NGS Reads	% Coverage	Closest Reference	NGS Reads	% Coverage	NGS Reads	% Coverage	Closest Reference	Total
NYB4466	USA-NY	2018	−1	29.41	−1	−1	3394	99%	ABT3406	2539	100%	2616	97%	ABT3406	8,048,648
NYB4468	USA-NY	2018	−1	33.36	−1	30.34	35	62%	ABT3406	28	70%	35	67%	ABT3406	14,782,666
NYB4138	USA-NY	2018	−1	26.4	−1	23.87	201	96%	ABT3406	143	97%	266	94%	ABT3406	7,321,340
MRN001	COL	2019	26.53	30.78	−1	24.06	178	100%	ABT3406	139	100%	288	97%	ABT3406	3,749,428
MRN006	COL	2019	22.18	28.33	−1	20.42	2229	100%	ABT3406	1655	100%	2905	100%	ABT3406	3,541,114
MRN012	COL	2019	−1	23.36	22.78	−1	111	42%	KY928792	111	50%	215	96%	KY928727	9,517,318
MRN012	COL	2019	−1	23.36	22.78	−1	3202	100%	MRN012	2190	100%	3020	100%	KM285233	9,517,318
MRN015	COL	2019	24.69	23.1	24.15	25.5	7518	100%	ABT3406	5425	100%	5793	99%	ABT3406	4,685,264
MRN015	COL	2019	24.69	23.1	24.15	25.5	15,340	100%	MRN012	10,584	100%	11,053	100%	KM285233	4,685,264
MRN016	COL	2019	−1	28.71	−1	25.56	675	100%	ABT3406	586	100%	812	100%	ABT3406	14,733,470
MRN020	COL	2019	−1	35.51	−1	−1	169	87%	ABT3406	131	89%	72	67%	ABT3406	34,621,664
MRN021	COL	2019	35.59	35.25	−1	31.49	36	54%	ABT3406	22	54%	72	92%	ABT3406	34,481,740
MRN023	COL	2019	−1	25.12	24.22	−1	3233	100%	MRN012	2533	100%	3925	100%	KM285233	8,746,944
MRN031	COL	2019	−1	32.26	−1	30.16	25	62%	MK204396	22	71%	20	47%	ABT3406	6,175,024
MRN032	COL	2019	−1	27.69	−1	25.94	66	92%	MK204396	56	89%	57	95%	ABT3406	5,853,846
MRN033	COL	2019	−1	25.35	26.91	34.56	28	48%	MK204396	14	40%	307	100%	MH933835	16,786,708
MRN033	COL	2019	−1	25.35	26.91	34.56	1880	99%	MRN012	1439	100%	1361	100%	KM285233	16,786,708
MRN034	COL	2019	−1	26.41	27.39	35.75	12	30%	MK204396	10	33%	957	100%	MH933835	9,571,150
MRN034	COL	2019	−1	26.41	27.39	35.75	2627	100%	MRN012	2071	100%	1315	100%	KM285233	9,571,150
MRN035	COL	2019	35.84	29.3	−1	26.64	458	100%	ABT3406	431	100%	437	92%	ABT3406	8,335,936
MRN038	COL	2019	26.57	30.67	−1	29.08	352	100%	ABT3406	300	100%	280	97%	ABT3406	1,585,992
MRN039	COL	2019	34.43	21.56	25.45	29.43	545	100%	MK204396	363	100%	417	89%	MH933835	4,737,986
MRN039	COL	2019	34.43	21.56	25.45	29.43	1416	97%	MRN012	1372	100%	2387	99%	KM285233	4,737,986
MRN044	COL	2019	22.58	31.48	−1	26.95	5395	99%	ABT3406	4823	98%	5603	100%	ABT3406	8,691,792
MRN044	COL	2019	22.58	31.48	−1	26.95	40	56%	MRN012	36	67%	109	70%	KM285233	8,691,792
MRN046	COL	2019	19.61	27.14	−1	23.29	2490	99%	ABT3406	1786	99%	2302	100%	ABT3406	4,679,714

Cts for each of four probes are listed, along with the number of NGS reads mapping for each strain, the percent genome coverage, and total NGS reads obtained.

**Table 2:** mNGS results and clinical data for PBV positives.

Patient ID	Age	Sex	RdRp FAM Ct	Log Titer cp/mL	Sputum Color	Viral Coinfection	Bacterial Coinfection	Medical Diagnosis	Symptoms
NYB4466	52	M	29.41	4.34	Yellow	HHV-4	Stenotrophomonas, Mycoplasma	n.d.	n.d.
NYB4468	40	F	33.36	3.15	Clear	Influenza, Strep phage	Streptococcus pneumonia, Bacillus	n.d.	n.d.
NYB4138	71	M	26.4	5.25	Brown/yellow	Klebsiella phage	Klebsiella pneumonia	n.d.	n.d.
NYB4477	51	F	36.26	2.28	Brown/yellow	none	None	n.d.	n.d.
NYB4470	62	F	34.6	2.23	Yellow	HHV-1, coronavirus OC43	None	n.d.	n.d.
BBL46722	57	F	35.04	2.10	Brown	None	None	n.d.	n.d.
MRN001	32	F	30.78	3.38	Brown	None	Treponema, Bacteroides	Myco TB+	Multidrug resistance, 2nd phase of treatment, fever, weight loss, chest pain, pain with breathing
MRN006	36	M	28.33	4.12	Clear	None	none	Pulmonary TB	Smear microscopy (+), BARR (++), chest pains, fever, weight loss, fatigue
MRN012	18	F	23.36	5.62	Clear	Respirovirus 3	none	Pulmonary TB	Smear microscopy (−), Radiography (+), chest pains, cough, fever, weight loss
Sptm Ctrl			36.34	1.71	n.d.	None	Klebsiella pneumonia	n.d.	n.d.
MRN015	36	M	23.1	5.96	Clear	None	Bordetalla, Burkholderia, Pseudomonas, Serretia	Pulmonary TB	Smear microscopy (+), BARR++, severe cough, fever, weight loss, chills, fatigue
MRN016	28	F	28.71	4.27	Clear	Pseudomonas phage	Pseudomonas aeruginosa	Pulmonary TB	Smear microscopy (+), BARR+++, 2nd phase of treatment
MRN020	83	M	35.51	2.22	Clear	None	Mycoplasma, Treponema, Streptococcus	Negative	Coughing up blood, fatigue, weight loss
MRN021	68	F	35.25	2.30	Yellow/clear	Enterovirus D	None	Negative	Fever, night sweats, pain when breathing
MRN023	60	M	25.12	5.35	Brown/yellow	None	Haemophilus parainfluenza	Negative	Night sweats, fever, coughing up blood
MRN031	86	M	32.26	3.20	Clear	Rhinovirus A	Clostridium	Smear(−), Rad(+)	Fatigue, fever, night sweats
MRN032	71	M	27.69	4.58	Yellow	Rhinovirus A, Haemophilus virus	Haemophilus influenzae	Pneumonia	Chest pains, coughing up blood, pain when breathing
MRN033	52	M	25.35	5.28	Clear	None	Bacillus, Treponema	Chronic bronchitis (−)	Chest pains, Chest pains when coughing
MRN034	61	M	26.41	4.96	Clear	None	Bacillus, Mycoplasma, Treponema	EPOC BARR(+)	Night sweats
MRN035	68	M	29.3	4.09	Clear	None	Spirochaeta, Treponema, Bacteroides, Mycoplasma	Pulmonary TB, Barr(++)	Coughing up blood, fatigue, chest pains
MRN036	71	F	34.19	2.62	Yellow	None	None	Bronchopneumonia (−)	Chest pains, hest pains when coughing
MRN038	69	M	30.67	3.68	Clear	None	Burkholderia, Serretia, Pseudomonas	EPOC BARR (−)	Fatigue, night sweats
MRN039	20	M	21.56	6.42	Clear	None	Treponema	Chronic bronchitis (−)	Chest pains when coughing and breathing
MRN044	72	M	31.48	3.44	Clear	None	None	EPOC BARR (−)	Night sweats, chills, fatigue
MRN046	72	F	27.14	4.74	Clear	None	None	Pneumonia (−)	Chest pains when coughing, weight loss, chills

Demographic data are included with estimated PBV titers (log copies/mL) based on RdRp FAM Cts. Viral and bacterial co-infections detected by SURPI enriched ≥10 fold are listed. Clinical diagnosis, symptoms, and sputum color tinge are listed for each.

## Data Availability

The complete genome sequences generated in this study were submitted to the GenBank database (https://www.ncbi.nlm.nih.gov/genbank/) accessed on 9 December 2021 under the following accessions: OL875303-OL875351. Raw data was deposited under BioProject number PRJNA789273 and SRA study SRP350910.

## Supplementary Materials

The following are available online at https://www.mdpi.com/article/10.3390/v13122534/s1, Methods: Primer and Probe sequences, RT-qPCR experimental conditions, Supplemental Figure S1: Maximum-Likelihood phylogenetic tree of PBV capsid, Supplemental Figure S2: RT-qPCR detection of ABT3406, Supplemental Figure S3: Aichivirus RT-qPCR assay, Supplemental Table S1: Listing of GenBank RDRP references, Supplemental Table S2: Listing of GenBank capsid references, Supplemental Table S3: Viral and bacterial infections detected in primary screen, Supplemental Table S4: Phylogeny-trait association tests for PBV RdRp and capsid coding sequences using BaTS.
