# Supplementary material for: Emergence of a Distinct Picobirnavirus Genotype Circulating in Patients Hospitalized with Acute Respiratory Illness
Source: Viruses. 2021 Dec 17;13(12):2534. doi: 10.3390/v13122534 (PMC8708096; doi:10.3390/v13122534)
Supplement: Supplementary file 1 [file viruses-13-02534-s001.zip › Supplementary_information.pdf]

Online Supplemental Appendix

A DISTINCT GENOTYPE OF PICOBIRNAVIRUSES CIRCULATING IN PATIENTS HOSPTIALIZED WITH ACUTE RESPIRATORY ILLNESS

Michael G. Berg, Kenn Forberg, Lester Perez, Ka-Cheung Luk, Todd V. Meyer, and Gavin A. Cloherty

Primer and Probe sequences

Capsid

Forward Primer: CAF1151: 5'-CACCTACTCCAGATGATGTC-3'

Reverse Primer: CAR1229: 5'-CTGTACCCATAGCAGTGAATA

Probe: CAP1186: 5' FAM-TTAGCTGTGGCATTAGAACCAGGCGC-BHQ1 3'

RDRP

Forward Primers:

(1) PVFP1: 5'-TGGCGIGGICARGAAGG-3'

(2) PVFP2: 5'-TGGAGAGGICAIGARGG-3'

(3) PVFP3: 5'-TGGCGIGGICARGAGGG-3'

Reverse Primers:

(1) PVRP1: 5'-CCATICIAAYCCAIGCAGG-3'

(2) PVRP2: 5'-CIAWGCIAACCCAIGCTGG-3'

(3) KMRP: 5'-CAIICCGACCCAWGCTGG-3'

(4) GQRP: 5'-ATAAACCAATCCATGGCGCTAT-3'

(5) MGRP: 5'-ACCICGTCATTRCIIWCCCA-3'

Probes:

(1) PVPROF1: 5' FAM-CGTIAARCARIGIGTIGTITGGATGTTYCC-BHQ1 3'

(2) PVPROF2: 5' FAM-CGTIAARCARAGIGTIGTITGGATGTTCCC-BHQ1 3'

(3) PVPROF3: 5' FAM-CGTIAARCAGCGIGTIGTITGGATGTTYCC-BHQ1 3'

(4) MRNRPRO: 5' Cy5-CGTTGCGCTGTTTCGATTAGAGGTTGG-BHQ2 3'

(5) KMRPRO: 5' Cy3-TGTAGCATATCCATAAACGGCTGRTAGAC-BHQ2 3'

PCR reactions and cycling conditions

Below is a detailed description of the PBV Capsid qPCR reaction recipe and cycling conditions:

Water	11.45 µl		
2X RT-PCR Buffer	25.0 µl (1X)		
CAF1151 (100 µM in TE, pH 8.0)	0.2 µl	0.55 µl	(0.4 µM)
CAR1229 (100 µM in TE, pH 8.0)	0.2 µl		(0.4 µM)
CAP1186 (FAM) (100 µM in TE, pH 8.0)	0.15 µl		(0.3 µM)
50 mM $MgCl_2$	1.0 µl (1 mM)		
25 x RT-PCR Enzyme Mix	2.0 µl (1X)		
	40 µl		
RNA	10 µl		
	50 µl per reaction		

Real-time PCR Cycling Conditions

Stage	Cycle	Temperature	Time
1	1	50 C	30 min
2	1	95 C	10 min
3	45	95 C	30 sec
		62 C	30 sec
		55 C	90 sec (signals read in last 30 sec)

Below is a detailed description of the PBV RDRP qPCR reaction recipe and cycling conditions:

Water	9.65 µl		
2 x RT-PCR Buffer	25.0 µl (1 x)		
PVFP1 (100 µM in TE, pH8.0)	0.2 µl	2.05 µl	(0.4 µM)
PVFP2 (100 µM in TE, pH 8.0)	0.2 µl		(0.4 µM)
PVFP3 (100 µM in TE, pH 8.0)	0.2 µl		(0.4 µM)
PVRP1 (100 µM in TE, pH 8.0)	0.2 µl		(0.4 µM)
PVRP2 (100 µM in TE, pH 8.0)	0.2 µl		(0.4 µM)
KMRP (100 µM in TE, pH 8.0)	0.2 µl		(0.4 µM)
GQRP (100 µM in TE, pH 8.0)	0.2 µl		(0.4 µM)
MGRP (100 µM in TE, pH 8.0)	0.2 µl		(0.4 µM)
PVPROF1 (FAM) (100 µM in TE, pH 8.0)	0.15 µl		(0.3 µM)
PVPROF2 (FAM) (100 µM in TE, pH 8.0)	0.15 µl		(0.3 µM)
PVPROF3 (FAM) (100 µM in TE, pH 8.0)	0.15 µl		(0.3 µM)
MRNRPRO (Cy5) (100 µM in TE, pH 7.0)	0.15 µl	0.3 µl	(0.3 µM)
KMRPRO (Cy3) (100 µM in TE, pH 7.0)	0.15 µl		(0.3 µM)
50 mM $MgCl_2$	1.0 µl (1 mM)		
25 x RT-PCR Enzyme Mix	2.0 µl (1 x)		
	40 µl		
RNA	10 µl		
	50 µl per reaction		

Real-time PCR Cycling Conditions

Stage	Cycle	Temperature	Time
1	1	50 C	30 min
2	1	95 C	10 min
3	45	95 C	30 sec
		62 C	30 sec
		55 C	90 sec (read signals in last 30 sec)

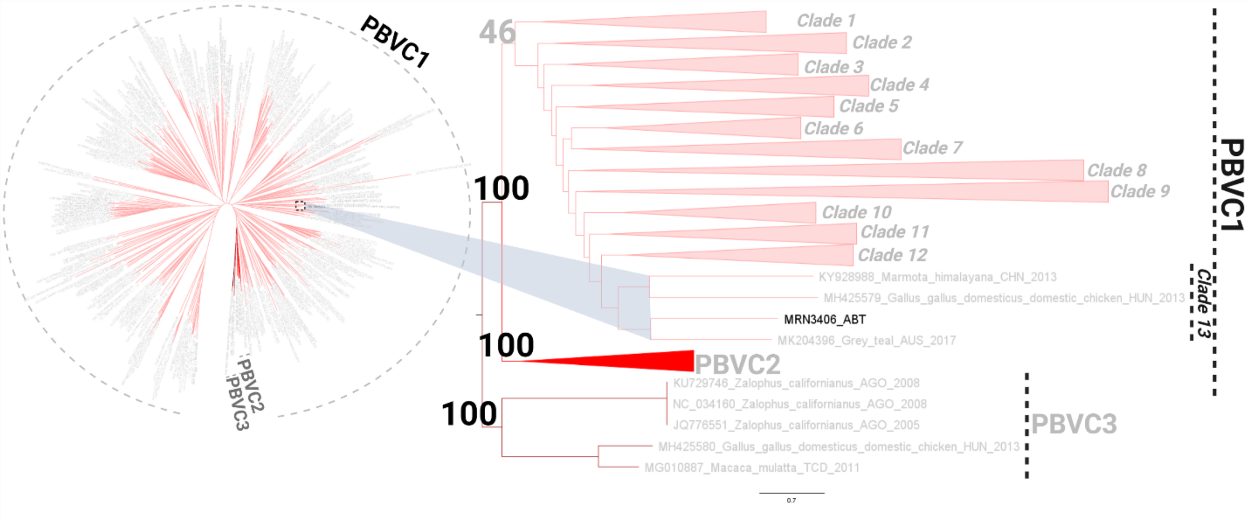

Supplemental Figure S1. Maximum-Likelihood phylogenetic radial tree (left) of 422 PBV capsid sequences retrieved from GenBank along with the index case. Species PBVC1 has been expanded, with clades therein collapsed for visualization. The MRN3406 ABT index strain capsid branches within clade 13 of species-PBVC1 along with MK204396, a grey teal duck from Australia.

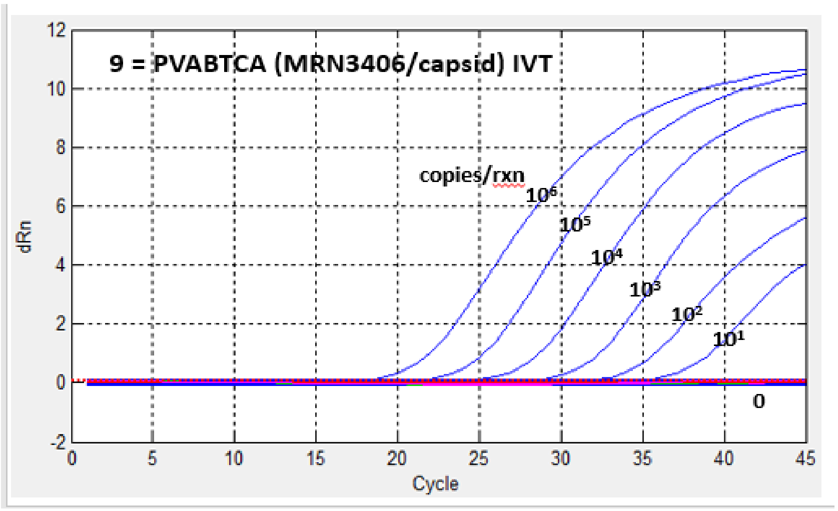

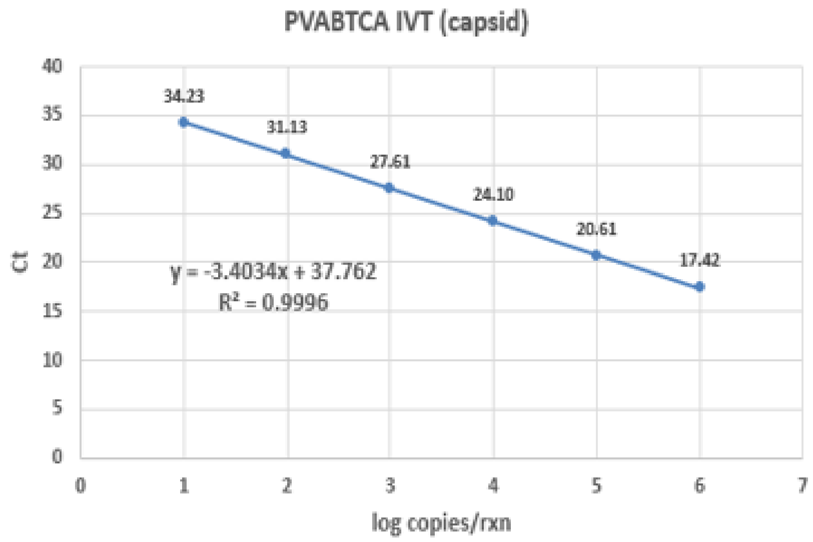

Supplemental Figure S2 (top) For detection of the ABT3406 capsid, *in vitro* transcript PVABTCA was resuspended in water at 10^1-10^6 copies/10 µl. Only one probe labeled with FAM detects capsid amplicons. (bottom) Linear regression analysis comparing Ct vs log copies/reaction.

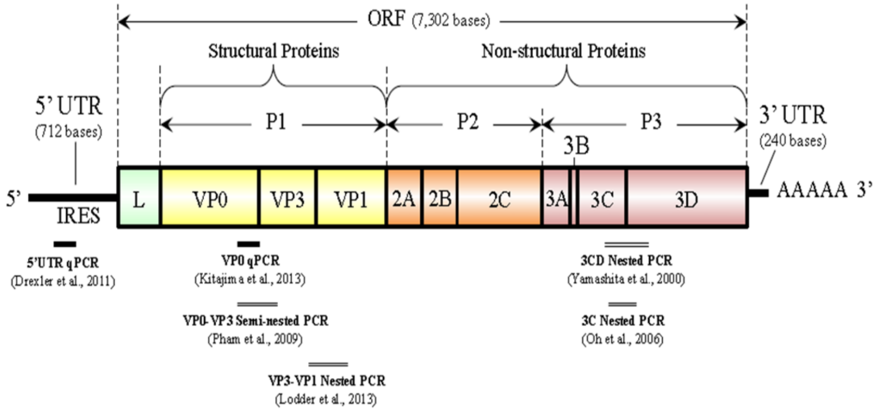

Aichivirus A genome organization and published PCR assays.

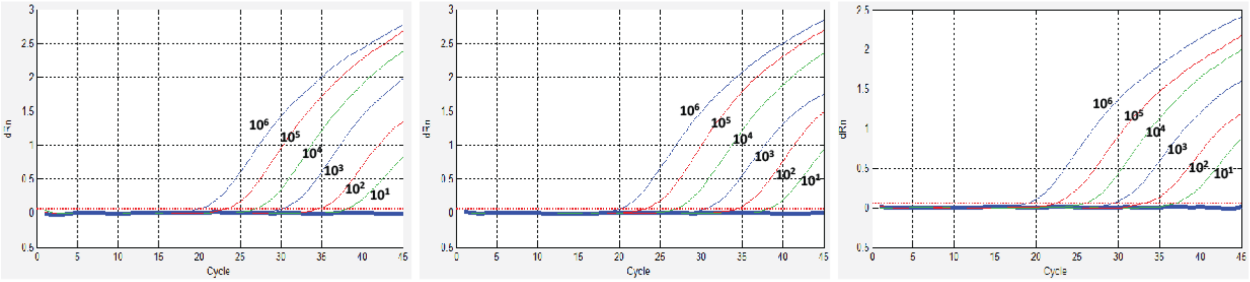

1 = AVABT (ABT4352) IVT
Genotype B

2 = AVDQ (DQ028632) IVT
Genotype B

3 = AVNC (NC_001918) IVT
Genotype A

Supplemental Figure S3. Sputum sample 9-4352 from the original MRN Dx panel was positive for Aichivirus A. We recovered 80% of the genome and its top BLAST hit was GenBank accession # NP_047200. An Aichivirus qPCR assay targeting the VP0 region was developed based on Kitajim, *et al*, 2013. Three separate IVTs were generated to ensure detection of the strain from ABT4352 and additional genotype B (DQ028632) and genotype A (NC_001918) strains. IVTs diluted from 10^1-10^6 copies/10 µl were detected with FAM labeled probes.

GenBank ID	Host	Country code	Years
AB186898	*NA*	NA	NA
AB517731	*NA*	IND	2007
AB517732	*NA*	IND	2007
AB517733	*NA*	IND	2007
AB517734	*NA*	IND	2007
AB517735	*NA*	IND	2007
AB517736	*NA*	IND	2007
AB517737	*NA*	IND	2007
AB517738	*NA*	IND	2007
AB517739	*NA*	IND	2008
AB828072	*Bos taurus*	CHN	NA
AF246939	*NA*	TCD	NA
AF246940	*NA*	NA	NA
GQ221268	*Bos indicus*	IND	2005
GQ915026	*Homo sapiens*	TCD	2004
GQ915029	*Homo sapiens*	TCD	NA
GU968924	*Homo sapiens*	NLD	2007
HM070240	*Sus scrofa*	CHN	2010
JF755419	*Mus musculus*	TCD	2008
JF755420	*Microtus pennsylvanicus*	TCD	2008
JQ710506	*Macaca mulatta*	CHN	2002
JQ710507	*Macaca mulatta*	CHN	2002
JQ776552	*Zalophus californianus*	AGO	2008
KC692366	*Vulpes vulpes*	NLD	2012
KF792838	*Felis catus*	PRT	2012
KF823810	*Vulpes vulpes*	ESP	2013
KF823811	*Vulpes vulpes*	ESP	2013
KF861773	*Sus scrofa*	ITA	2004
KJ206569	*Homo sapiens*	NLD	NA
KJ495690	*Meleagris gallopavo*	TCD	2011
KJ663814	*Homo sapiens*	TCD	2013
KJ663816	*Homo sapiens*	TCD	2013
KM254161	*Gallus gallus*	ZAF	2013
KM254162	*Gallus gallus*	ZAF	2013
KM254164	*Gallus gallus*	ZAF	2013
KM285233	*Homo sapiens*	KHM	2009
KM285234	*Homo sapiens*	KHM	2009
KM573798	*Camelus dromedarius*	ARE	2013

KM573799	*Camelus dromedarius*	ARE	2013
KM573800	*Camelus dromedarius*	ARE	2013
KM573801	*Camelus dromedarius*	ARE	2013
KM573802	*Camelus dromedarius*	ARE	2013
KM573803	*Camelus dromedarius*	ARE	2013
KM573804	*Camelus dromedarius*	ARE	2013
KM573805	*Camelus dromedarius*	ARE	2013
KM573806	*Camelus dromedarius*	ARE	2013
KM573807	*Camelus dromedarius*	ARE	2013
KM573808	*Camelus dromedarius*	ARE	2013
KM573809	*Camelus dromedarius*	ARE	2013
KP941111	*Vulpes vulpes*	HRV	2014
KP984805	*Sus scrofa*	CHN	2012
KR106195	*Arctocephalus australis*	BRA	2012
KR827412	*Homo sapiens*	CHN	2012
KR827413	*Homo sapiens*	CHN	2012
KR827414	*Homo sapiens*	CHN	2012
KR827415	*Homo sapiens*	CHN	2013
KR827416	*Homo sapiens*	CHN	2013
KR827417	*Homo sapiens*	CHN	2014
KR827418	*Homo sapiens*	CHN	2013
KR902502	*Equus caballus*	TCD	2012
KR902503	*Equus caballus*	TCD	2012
KR902505	*Equus caballus*	TCD	2012
KR902507	*Equus caballus*	TCD	2012
KT934307	*Canis lupus*	PRT	2015
KT934308	*Canis lupus*	PRT	2015
KT984499	*Macaca mulatta*	TCD	2014
KU729755	*Zalophus californianus*	AGO	2008
KU729756	*Zalophus californianus*	AGO	2008
KU729757	*Zalophus californianus*	AGO	2008
KU729758	*Zalophus californianus*	AGO	2008
KU729759	*Zalophus californianus*	AGO	2008
KU729760	*Zalophus californianus*	AGO	2008
KU729761	*Zalophus californianus*	AGO	2009
KU729762	*Zalophus californianus*	AGO	2009
KU729763	*Zalophus californianus*	AGO	2009
KU729764	*Zalophus californianus*	AGO	2009
KU729765	*Zalophus californianus*	AGO	2009

KU729766	*Zalophus californianus*	AGO	2009
KU729767	*Zalophus californianus*	AGO	2009
KU729768	*Zalophus californianus*	AGO	2010
KU729769	*Zalophus californianus*	AGO	2010
KU892528	*Homo sapiens*	BEL	2010
KU892529	*Homo sapiens*	BEL	2010
KU892530	*Homo sapiens*	BEL	2010
KX374476	*Bos taurus*	IND	2015
KX374477	*Sus scrofa*	IND	2013
KX374478	*Sus scrofa*	IND	2013
KY053140	*Chlorocebus sabaeus*	KNA	2015
KY053141	*Chlorocebus sabaeus*	KNA	2015
KY053142	*Chlorocebus sabaeus*	KNA	2015
KY053143	*Chlorocebus sabaeus*	KNA	2015
KY120170	*Felis catus*	AGO	2012
KY120171	*Felis catus*	AGO	2012
KY120172	*Felis catus*	AGO	2012
KY120173	*Felis catus*	AGO	2012
KY120174	*Felis catus*	AGO	2012
KY120175	*Felis catus*	AGO	2012
KY120176	*Felis catus*	AGO	2012
KY120177	*Felis catus*	AGO	2012
KY120178	*Felis catus*	AGO	2012
KY120179	*Felis catus*	AGO	2012
KY120180	*Felis catus*	AGO	2012
KY120181	*Felis catus*	AGO	2012
KY120182	*Simiiformes*	AGO	2009
KY120183	*Simiiformes*	AGO	2009
KY120184	*Simiiformes*	AGO	2009
KY120185	*Simiiformes*	AGO	2009
KY120186	*Simiiformes*	AGO	2009
KY120187	*Simiiformes*	AGO	2009
KY120188	*Simiiformes*	AGO	2009
KY120189	*Simiiformes*	AGO	2009
KY120190	*Simiiformes*	AGO	2009
KY120191	*Simiiformes*	AGO	2009
KY120192	*Simiiformes*	AGO	2009
KY120193	*Simiiformes*	AGO	2009
KY120194	*Simiiformes*	AGO	2009

KY174983	*Macaca mulatta*	TCD	2012
KY214430	*Sus scrofa*	BEL	2015
KY214431	*Sus scrofa*	BEL	2015
KY214432	*Sus scrofa*	BEL	2015
KY399057	*Canis lupus*	KNA	2015
KY502850	*Gorilla gorilla*	COD	2015
KY502851	*Gorilla gorilla*	COD	2015
KY502852	*Gorilla gorilla*	COD	2015
KY502853	*Gorilla gorilla*	COD	2015
KY502854	*Gorilla gorilla*	COD	2015
KY502855	*Gorilla gorilla*	COD	2015
KY502856	*Gorilla gorilla*	COD	2015
KY502862	*Gorilla gorilla*	COD	2015
KY502863	*Gorilla gorilla*	COD	2015
KY502864	*Gorilla gorilla*	COD	2015
KY502865	*Gorilla gorilla*	COD	2015
KY502866	*Gorilla gorilla*	COD	2015
KY502867	*Gorilla gorilla*	COD	2015
KY502868	*Gorilla gorilla*	COD	2015
KY502869	*Gorilla gorilla*	COD	2015
KY502870	*Gorilla gorilla*	COD	2015
KY502871	*Gorilla gorilla*	COD	2015
KY502872	*Gorilla gorilla*	COD	2015
KY502873	*Gorilla gorilla*	COD	2015
KY502874	*Gorilla gorilla*	COD	2015
KY502875	*Gorilla gorilla*	COD	2015
KY502876	*Gorilla gorilla*	COD	2015
KY502879	*Gorilla gorilla*	COD	2015
KY502961	*Gorilla gorilla*	COD	2015
KY502962	*Gorilla gorilla*	COD	2015
KY502963	*Gorilla gorilla*	COD	2015
KY502964	*Gorilla gorilla*	COD	2015
KY502965	*Gorilla gorilla*	COD	2015
KY502966	*Gorilla gorilla*	COD	2015
KY502967	*Gorilla gorilla*	COD	2015
KY502968	*Gorilla gorilla*	COD	2015
KY502969	*Gorilla gorilla*	COD	2015
KY502970	*Gorilla gorilla*	COD	2015
KY502978	*Gorilla gorilla*	COD	2015

KY502979	*Gorilla gorilla*	COD	2015
KY502980	*Gorilla gorilla*	COD	2015
KY502982	*Gorilla gorilla*	COD	2015
KY502985	*Gorilla gorilla*	COD	2015
KY502986	*Gorilla gorilla*	COD	2015
KY502987	*Gorilla gorilla*	COD	2015
KY502988	*Gorilla gorilla*	COD	2015
KY502989	*Gorilla gorilla*	COD	2015
KY502990	*Gorilla gorilla*	COD	2015
KY502991	*Gorilla gorilla*	COD	2015
KY502992	*Gorilla gorilla*	COD	2015
KY502993	*Gorilla gorilla*	COD	2015
KY502994	*Gorilla gorilla*	COD	2015
KY502996	*Gorilla gorilla*	COD	2015
KY502998	*Gorilla gorilla*	COD	2015
KY502999	*Gorilla gorilla*	COD	2015
KY503001	*Gorilla gorilla*	COD	2015
KY503004	*Gorilla gorilla*	COD	2015
KY503005	*Gorilla gorilla*	COD	2015
KY503009	*Gorilla gorilla*	COD	2015
KY503010	*Gorilla gorilla*	COD	2015
KY503020	*Gorilla gorilla*	COD	2015
KY855428	*Marmota himalayana*	CHN	2013
KY855429	*Marmota himalayana*	CHN	2013
KY855430	*Marmota himalayana*	CHN	2013
KY855431	*Marmota himalayana*	CHN	2013
KY928683	*Marmota himalayana*	CHN	2013
KY928684	*Marmota himalayana*	CHN	2013
KY928685	*Marmota himalayana*	CHN	2013
KY928686	*Marmota himalayana*	CHN	2013
KY928687	*Marmota himalayana*	CHN	2013
KY928688	*Marmota himalayana*	CHN	2013
KY928689	*Marmota himalayana*	CHN	2013
KY928690	*Marmota himalayana*	CHN	2013
KY928691	*Marmota himalayana*	CHN	2013
KY928692	*Marmota himalayana*	CHN	2013
KY928693	*Marmota himalayana*	CHN	2013
KY928694	*Marmota himalayana*	CHN	2013
KY928695	*Marmota himalayana*	CHN	2013

KY928696	*Marmota himalayana*	CHN	2013
KY928697	*Marmota himalayana*	CHN	2013
KY928698	*Marmota himalayana*	CHN	2013
KY928699	*Marmota himalayana*	CHN	2013
KY928700	*Marmota himalayana*	CHN	2013
KY928701	*Marmota himalayana*	CHN	2013
KY928702	*Marmota himalayana*	CHN	2013
KY928703	*Marmota himalayana*	CHN	2013
KY928704	*Marmota himalayana*	CHN	2013
KY928705	*Marmota himalayana*	CHN	2013
KY928706	*Marmota himalayana*	CHN	2013
KY928707	*Marmota himalayana*	CHN	2013
KY928708	*Marmota himalayana*	CHN	2013
KY928709	*Marmota himalayana*	CHN	2013
KY928710	*Marmota himalayana*	CHN	2013
KY928711	*Marmota himalayana*	CHN	2013
KY928712	*Marmota himalayana*	CHN	2013
KY928713	*Marmota himalayana*	CHN	2013
KY928714	*Marmota himalayana*	CHN	2013
KY928715	*Marmota himalayana*	CHN	2013
KY928716	*Marmota himalayana*	CHN	2013
KY928717	*Marmota himalayana*	CHN	2013
KY928718	*Marmota himalayana*	CHN	2013
KY928719	*Marmota himalayana*	CHN	2013
KY928720	*Marmota himalayana*	CHN	2013
KY928721	*Marmota himalayana*	CHN	2013
KY928722	*Marmota himalayana*	CHN	2013
KY928723	*Marmota himalayana*	CHN	2013
KY928724	*Marmota himalayana*	CHN	2013
KY928725	*Marmota himalayana*	CHN	2013
KY928726	*Marmota himalayana*	CHN	2013
KY928727	*Marmota himalayana*	CHN	2013
KY928728	*Marmota himalayana*	CHN	2013
KY928729	*Marmota himalayana*	CHN	2013
KY928730	*Marmota himalayana*	CHN	2013
KY928731	*Marmota himalayana*	CHN	2013
KY928732	*Marmota himalayana*	CHN	2013
KY928733	*Marmota himalayana*	CHN	2013
KY928734	*Marmota himalayana*	CHN	2013

KY928735	*Marmota himalayana*	CHN	2013
KY928736	*Marmota himalayana*	CHN	2013
KY928737	*Marmota himalayana*	CHN	2013
KY928738	*Marmota himalayana*	CHN	2013
LC110353	*Mus musculus*	JPN	2015
LC338002	*Camelus dromedarius*	ARE	2013
LC338003	*Camelus dromedarius*	ARE	2013
LC338004	*Camelus dromedarius*	ARE	2013
LC338005	*Camelus dromedarius*	ARE	2013
LC338006	*Camelus dromedarius*	ARE	2013
LC338007	*Camelus dromedarius*	ARE	2013
LC338008	*Camelus dromedarius*	ARE	2013
LC338009	*Camelus dromedarius*	ARE	2013
MF071281	*Felis catus*	KNA	2014
MF416389	*Mus musculus*	TCD	2015
MF416390	*Mus musculus*	TCD	2014
MF416391	*Mus musculus*	TCD	2014
MG003334	*Camelus dromedarius*	IND	2016
MG003339	*Bos taurus*	IND	2015
MG003340	*Bos taurus*	IND	2015
MG003341	*Bos taurus*	IND	2015
MG010904	*Macaca mulatta*	TCD	2011
MG010905	*Macaca mulatta*	TCD	2011
MG010906	*Macaca mulatta*	TCD	2011
MG010907	*Macaca mulatta*	TCD	2011
MG010908	*Macaca mulatta*	TCD	2011
MG010909	*Macaca mulatta*	TCD	2011
MG010910	*Macaca mulatta*	TCD	2011
MG010911	*Macaca mulatta*	TCD	2011
MG010912	*Macaca mulatta*	TCD	2011
MG010913	*Macaca mulatta*	TCD	2011
MG010915	*Macaca mulatta*	TCD	2011
MG010916	*Macaca mulatta*	TCD	2011
MG010917	*Macaca mulatta*	TCD	2011
MG010918	*Macaca mulatta*	TCD	2011
MG010919	*Macaca mulatta*	TCD	2011
MG010920	*Macaca mulatta*	TCD	2011
MG010921	*Macaca mulatta*	TCD	2011
MG190029	*Roe deer*	SVN	2014

MG571903	*Homo sapiens*	SEN	2015
MG571907	*Homo sapiens*	SEN	2015
MG600063	*Parupeneus cyclostomus*	CHN	NA
MG600064	*Tropidophorus sinicus*	CHN	NA
MG821233	*Caprine*	IND	2015
MG846401	*Gallus gallus*	BRA	2015
MG846402	*Gallus gallus*	BRA	2015
MG846403	*Gallus gallus*	BRA	2015
MG846404	*Gallus gallus*	BRA	2015
MG846405	*Gallus gallus*	BRA	2015
MG846406	*Gallus gallus*	BRA	2015
MG846407	*Gallus gallus*	BRA	2015
MG846408	*Gallus gallus*	BRA	2015
MG846409	*Gallus gallus*	BRA	2015
MG846410	*Gallus gallus*	BRA	2015
MG846411	*Gallus gallus*	BRA	2015
MG846412	*Gallus gallus*	BRA	2015
MH327934	*Gallus gallus*	HUN	2011
MH412924	*Rattus sp.*	KNA	2017
MH453875	*Australian Shelduck*	AUS	2012
MH453878	*Australian Shelduck*	AUS	2012
MH933801	*Homo sapiens*	CMR	2014
MH933802	*Homo sapiens*	CMR	2014
MH933803	*Homo sapiens*	CMR	2014
MH933804	*Homo sapiens*	CMR	2014
MH933805	*Homo sapiens*	CMR	2014
MH933806	*Homo sapiens*	CMR	2014
MH933807	*Homo sapiens*	CMR	2014
MH933808	*Homo sapiens*	CMR	2014
MH933809	*Homo sapiens*	CMR	2014
MH933810	*Homo sapiens*	CMR	2014
MH933811	*Homo sapiens*	CMR	2014
MH933812	*Homo sapiens*	CMR	2014
MH933813	*Homo sapiens*	CMR	2014
MH933814	*Homo sapiens*	CMR	2014
MH933815	*Homo sapiens*	CMR	2014
MH933817	*Homo sapiens*	CMR	2014
MH933818	*Homo sapiens*	CMR	2014
MH933819	*Homo sapiens*	CMR	2014

MH933820	*Homo sapiens*	CMR	2014
MH933821	*Homo sapiens*	CMR	2014
MH933822	*Homo sapiens*	CMR	2014
MH933823	*Homo sapiens*	CMR	2014
MH933824	*Homo sapiens*	CMR	2014
MH933825	*Homo sapiens*	CMR	2014
MH933830	*Homo sapiens*	CMR	2014
MH933831	*Homo sapiens*	CMR	2014
MH933832	*Homo sapiens*	CMR	2014
MH933833	*Homo sapiens*	CMR	2014
MH933834	*Homo sapiens*	CMR	2014
MH933835	*Homo sapiens*	CMR	2014
MH933836	*Homo sapiens*	CMR	2014
MH933839	*Homo sapiens*	CMR	2014
MH933841	*Homo sapiens*	CMR	2014
MK064212	*Chiroptera*	CHN	2016
MK064213	*Chiroptera*	CHN	2016
MK204395	*Anas gracilis*	AUS	2017
MK204418	*Malacorhynchus membranaceus*	AUS	2017
MK305310	*Homo sapiens*	AGO	2018
MK378834	*Sus scrofa*	CHN	2017
MK378835	*Sus scrofa*	CHN	2017
MK378843	*Sus scrofa*	CHN	2017
MK378844	*Sus scrofa*	CHN	2017
MK378845	*Sus scrofa*	CHN	2017
MK378851	*Sus scrofa*	CHN	2017
MK378856	*Sus scrofa*	CHN	2017
MK378859	*Sus scrofa*	CHN	2017
MK378860	*Sus scrofa*	CHN	2017
MK378865	*Sus scrofa*	CHN	2017
MK378866	*Sus scrofa*	CHN	2017
MK378867	*Sus scrofa*	CHN	2017
MK378868	*Sus scrofa*	CHN	2017
MK378869	*Sus scrofa*	CHN	2017
MK378870	*Sus scrofa*	CHN	2017
MK378876	*Sus scrofa*	CHN	2017
MK521919	*Sarcophilus harrisii*	AUS	2017
MK521920	*Sarcophilus harrisii*	AUS	2016
MK521921	*Sarcophilus harrisii*	AUS	2017

MK521922	*Sarcophilus harrisii*	AUS	2017
MK521923	*Sarcophilus harrisii*	AUS	2017
MK521924	*Sarcophilus harrisii*	AUS	2016
MK521925	*Sarcophilus harrisii*	AUS	2016
MK521926	*Sarcophilus harrisii*	AUS	2017
MN145873	*Homo sapiens*	CHN	2018
MN563295	*Urva auropunctata*	KNA	2017
MN563296	*Urva auropunctata*	KNA	2017
MN563297	*Urva auropunctata*	KNA	2017
MN563298	*Urva auropunctata*	KNA	2017
MN563299	*Urva auropunctata*	KNA	2017
MN563300	*Urva auropunctata*	KNA	2017
MN563301	*Urva auropunctata*	KNA	2017
MN563302	*Urva auropunctata*	KNA	2017
MN692671	*Macaca fascicularis*	THA	2017
MN871976	*Chlorocebus sabaeus*	CHN	2018
MT129742	*NA*	AUS	2017
MT129743	*NA*	AUS	2017
MT129744	*NA*	AUS	2017
MT129745	*NA*	AUS	2017
MT129746	*NA*	AUS	2017
MT129747	*NA*	AUS	2017
MT129748	*NA*	AUS	2017
MT129749	*NA*	AUS	2017
MT129750	*NA*	AUS	2017
MT129751	*NA*	AUS	2017
MT129752	*NA*	AUS	2017
MT129753	*NA*	AUS	2017
MT150089	*NA*	NA	2018
MT341487	*Actinonaias pectorosa*	TCD	2018
MT350351	*Pan troglodytes*	SLE	NA
MT350352	*Pan troglodytes*	SLE	NA
MT846991	*Pantholops hodgsonii*	CHN	2014
MT847000	*Pantholops hodgsonii*	CHN	2014
MT847001	*Pantholops hodgsonii*	CHN	2014
MT847002	*Pantholops hodgsonii*	CHN	2014
MT847003	*Pantholops hodgsonii*	CHN	2014
MT847004	*Pantholops hodgsonii*	CHN	2014
MT847005	*Pantholops hodgsonii*	CHN	2014

MT847006	*Pantholops hodgsonii*	CHN	2014
MT847007	*Pantholops hodgsonii*	CHN	2014
MT847008	*Pantholops hodgsonii*	CHN	2014
MT847009	*Pantholops hodgsonii*	CHN	2014
MT847010	*Pantholops hodgsonii*	CHN	2014
MT847011	*Pantholops hodgsonii*	CHN	2014
MT847012	*Pantholops hodgsonii*	CHN	2014
NC_007027	*NA*	THA	NA
NC_29802	*Sus scrofa*	ITA	2004
NC_34161	*Zalophus californianus*	AGO	2008
NC_34452	*Chlorocebus sabaeus*	KNA	2015
NC_35206	*Canis lupus*	KNA	2015
NC_40439	*Gallus gallus*	HUN	2011
NC_40753	*Roe deer*	SVN	2014

Supplemental Table S1. RdRp sequences of PBV used in the current study for phylogenetic analysis.

GenBank ID	Host	Country code	Years
JQ776551	*Zalophus californianus*	AGO	2005
KF861768	*Sus scrofa*	ITA	2004
KF861770	*Sus scrofa*	ITA	2004
KF861771	*Sus scrofa*	ITA	2004
KF861772	*Sus scrofa*	ITA	2004
KJ206568	*Homo sapiens*	NLD	NA
KJ495689	*Meleagris gallopavo*	TCD	2011
KJ663815	*Homo sapiens*	TCD	2013
KM573778	*Camelus dromedarius*	ARE	2013
KM573779	*Camelus dromedarius*	ARE	2013
KM573780	*Camelus dromedarius*	ARE	2013
KM573781	*Camelus dromedarius*	ARE	2013
KM573782	*Camelus dromedarius*	ARE	2013
KM573783	*Camelus dromedarius*	ARE	2013
KM573784	*Camelus dromedarius*	ARE	2013
KM573785	*Camelus dromedarius*	ARE	2013
KM573786	*Camelus dromedarius*	ARE	2013

KM573787	*Camelus dromedarius*	ARE	2013
KM573788	*Camelus dromedarius*	ARE	2013
KM573789	*Camelus dromedarius*	ARE	2013
KM573790	*Camelus dromedarius*	ARE	2013
KM573791	*Camelus dromedarius*	ARE	2013
KM573792	*Camelus dromedarius*	ARE	2013
KM573794	*Camelus dromedarius*	ARE	2013
KM573795	*Camelus dromedarius*	ARE	2013
KM573796	*Camelus dromedarius*	ARE	2013
KM573797	*Camelus dromedarius*	ARE	2013
KR902502	*Equus caballus*	TCD	2012
KR902504	*Equus caballus*	TCD	2012
KR902506	*Equus caballus*	TCD	2012
KR902508	*Equus caballus*	TCD	2012
KT934309	*Canis lupus*	PRT	2015
KT934310	*Canis lupus*	PRT	2015
KU729746	*Zalophus californianus*	AGO	2008
KU729747	*Zalophus californianus*	AGO	2009
KU729748	*Zalophus californianus*	AGO	2009
KU729749	*Zalophus californianus*	AGO	2008
KU729750	*Zalophus californianus*	AGO	2009
KU729751	*Zalophus californianus*	AGO	2009
KU729752	*Zalophus californianus*	AGO	2009
KU729753	*Zalophus californianus*	AGO	2009
KU729754	*Zalophus californianus*	AGO	2008
KU892524	*Homo sapiens*	BEL	2010
KU892525	*Homo sapiens*	BEL	2010
KU892526	*Homo sapiens*	BEL	2010
KU892527	*Homo sapiens*	BEL	2010
KY174982	*Macaca mulatta*	TCD	2012
KY214426	*Sus scrofa*	BEL	2015
KY214427	*Sus scrofa*	BEL	2015
KY214428	*Sus scrofa*	BEL	2015
KY214429	*Sus scrofa*	BEL	2015
KY502835	*Gorilla gorilla*	COD	2015
KY502836	*Gorilla gorilla*	COD	2015
KY502837	*Gorilla gorilla*	COD	2015
KY502838	*Gorilla gorilla*	COD	2015
KY502839	*Gorilla gorilla*	COD	2015

KY502840	*Gorilla gorilla*	COD	2015
KY502841	*Gorilla gorilla*	COD	2015
KY502842	*Gorilla gorilla*	COD	2015
KY502843	*Gorilla gorilla*	COD	2015
KY502845	*Gorilla gorilla*	COD	2015
KY502846	*Gorilla gorilla*	COD	2015
KY502847	*Gorilla gorilla*	COD	2015
KY502848	*Gorilla gorilla*	COD	2015
KY502849	*Gorilla gorilla*	COD	2015
KY502857	*Gorilla gorilla*	COD	2015
KY502858	*Gorilla gorilla*	COD	2015
KY502859	*Gorilla gorilla*	COD	2015
KY502860	*Gorilla gorilla*	COD	2015
KY502878	*Gorilla gorilla*	COD	2015
KY502932	*Gorilla gorilla*	COD	2015
KY502935	*Gorilla gorilla*	COD	2015
KY502937	*Gorilla gorilla*	COD	2015
KY502943	*Gorilla gorilla*	COD	2015
KY502944	*Gorilla gorilla*	COD	2015
KY502946	*Gorilla gorilla*	COD	2015
KY502947	*Gorilla gorilla*	COD	2015
KY502948	*Gorilla gorilla*	COD	2015
KY502951	*Gorilla gorilla*	COD	2015
KY502952	*Gorilla gorilla*	COD	2015
KY502953	*Gorilla gorilla*	COD	2015
KY502972	*Gorilla gorilla*	COD	2015
KY502973	*Gorilla gorilla*	COD	2015
KY502975	*Gorilla gorilla*	COD	2015
KY502977	*Gorilla gorilla*	COD	2015
KY503014	*Gorilla gorilla*	COD	2015
KY855428	*Marmota himalayana*	CHN	2013
KY855429	*Marmota himalayana*	CHN	2013
KY855430	*Marmota himalayana*	CHN	2013
KY855431	*Marmota himalayana*	CHN	2013
KY928739	*Marmota himalayana*	CHN	2013
KY928740	*Marmota himalayana*	CHN	2013
KY928741	*Marmota himalayana*	CHN	2013
KY928742	*Marmota himalayana*	CHN	2013
KY928743	*Marmota himalayana*	CHN	2013

KY928744	*Marmota himalayana*	CHN	2013
KY928745	*Marmota himalayana*	CHN	2013
KY928746	*Marmota himalayana*	CHN	2013
KY928747	*Marmota himalayana*	CHN	2013
KY928748	*Marmota himalayana*	CHN	2013
KY928749	*Marmota himalayana*	CHN	2013
KY928750	*Marmota himalayana*	CHN	2013
KY928751	*Marmota himalayana*	CHN	2013
KY928752	*Marmota himalayana*	CHN	2013
KY928753	*Marmota himalayana*	CHN	2013
KY928754	*Marmota himalayana*	CHN	2013
KY928755	*Marmota himalayana*	CHN	2013
KY928756	*Marmota himalayana*	CHN	2013
KY928757	*Marmota himalayana*	CHN	2013
KY928758	*Marmota himalayana*	CHN	2013
KY928759	*Marmota himalayana*	CHN	2013
KY928760	*Marmota himalayana*	CHN	2013
KY928761	*Marmota himalayana*	CHN	2013
KY928762	*Marmota himalayana*	CHN	2013
KY928763	*Marmota himalayana*	CHN	2013
KY928764	*Marmota himalayana*	CHN	2013
KY928765	*Marmota himalayana*	CHN	2013
KY928766	*Marmota himalayana*	CHN	2013
KY928767	*Marmota himalayana*	CHN	2013
KY928768	*Marmota himalayana*	CHN	2013
KY928769	*Marmota himalayana*	CHN	2013
KY928770	*Marmota himalayana*	CHN	2013
KY928771	*Marmota himalayana*	CHN	2013
KY928772	*Marmota himalayana*	CHN	2013
KY928773	*Marmota himalayana*	CHN	2013
KY928774	*Marmota himalayana*	CHN	2013
KY928775	*Marmota himalayana*	CHN	2013
KY928776	*Marmota himalayana*	CHN	2013
KY928777	*Marmota himalayana*	CHN	2013
KY928778	*Marmota himalayana*	CHN	2013
KY928779	*Marmota himalayana*	CHN	2013
KY928780	*Marmota himalayana*	CHN	2013
KY928781	*Marmota himalayana*	CHN	2013
KY928782	*Marmota himalayana*	CHN	2013

KY928783	*Marmota himalayana*	CHN	2013
KY928784	*Marmota himalayana*	CHN	2013
KY928785	*Marmota himalayana*	CHN	2013
KY928786	*Marmota himalayana*	CHN	2013
KY928787	*Marmota himalayana*	CHN	2013
KY928788	*Marmota himalayana*	CHN	2013
KY928789	*Marmota himalayana*	CHN	2013
KY928790	*Marmota himalayana*	CHN	2013
KY928791	*Marmota himalayana*	CHN	2013
KY928792	*Marmota himalayana*	CHN	2013
KY928793	*Marmota himalayana*	CHN	2013
KY928794	*Marmota himalayana*	CHN	2013
KY928795	*Marmota himalayana*	CHN	2013
KY928796	*Marmota himalayana*	CHN	2013
KY928797	*Marmota himalayana*	CHN	2013
KY928798	*Marmota himalayana*	CHN	2013
KY928799	*Marmota himalayana*	CHN	2013
KY928800	*Marmota himalayana*	CHN	2013
KY928801	*Marmota himalayana*	CHN	2013
KY928802	*Marmota himalayana*	CHN	2013
KY928803	*Marmota himalayana*	CHN	2013
KY928804	*Marmota himalayana*	CHN	2013
KY928805	*Marmota himalayana*	CHN	2013
KY928806	*Marmota himalayana*	CHN	2013
KY928807	*Marmota himalayana*	CHN	2013
KY928808	*Marmota himalayana*	CHN	2013
KY928809	*Marmota himalayana*	CHN	2013
KY928810	*Marmota himalayana*	CHN	2013
KY928811	*Marmota himalayana*	CHN	2013
KY928812	*Marmota himalayana*	CHN	2013
KY928813	*Marmota himalayana*	CHN	2013
KY928814	*Marmota himalayana*	CHN	2013
KY928815	*Marmota himalayana*	CHN	2013
KY928816	*Marmota himalayana*	CHN	2013
KY928817	*Marmota himalayana*	CHN	2013
KY928818	*Marmota himalayana*	CHN	2013
KY928819	*Marmota himalayana*	CHN	2013
KY928820	*Marmota himalayana*	CHN	2013
KY928821	*Marmota himalayana*	CHN	2013

KY928822	*Marmota himalayana*	CHN	2013
KY928823	*Marmota himalayana*	CHN	2013
KY928824	*Marmota himalayana*	CHN	2013
KY928825	*Marmota himalayana*	CHN	2013
KY928826	*Marmota himalayana*	CHN	2013
KY928827	*Marmota himalayana*	CHN	2013
KY928828	*Marmota himalayana*	CHN	2013
KY928829	*Marmota himalayana*	CHN	2013
KY928830	*Marmota himalayana*	CHN	2013
KY928831	*Marmota himalayana*	CHN	2013
KY928832	*Marmota himalayana*	CHN	2013
KY928833	*Marmota himalayana*	CHN	2013
KY928834	*Marmota himalayana*	CHN	2013
KY928835	*Marmota himalayana*	CHN	2013
KY928836	*Marmota himalayana*	CHN	2013
KY928837	*Marmota himalayana*	CHN	2013
KY928838	*Marmota himalayana*	CHN	2013
KY928839	*Marmota himalayana*	CHN	2013
KY928840	*Marmota himalayana*	CHN	2013
KY928841	*Marmota himalayana*	CHN	2013
KY928842	*Marmota himalayana*	CHN	2013
KY928843	*Marmota himalayana*	CHN	2013
KY928844	*Marmota himalayana*	CHN	2013
KY928845	*Marmota himalayana*	CHN	2013
KY928846	*Marmota himalayana*	CHN	2013
KY928848	*Marmota himalayana*	CHN	2013
KY928849	*Marmota himalayana*	CHN	2013
KY928850	*Marmota himalayana*	CHN	2013
KY928851	*Marmota himalayana*	CHN	2013
KY928852	*Marmota himalayana*	CHN	2013
KY928853	*Marmota himalayana*	CHN	2013
KY928854	*Marmota himalayana*	CHN	2013
KY928855	*Marmota himalayana*	CHN	2013
KY928856	*Marmota himalayana*	CHN	2013
KY928857	*Marmota himalayana*	CHN	2013
KY928858	*Marmota himalayana*	CHN	2013
KY928859	*Marmota himalayana*	CHN	2013
KY928860	*Marmota himalayana*	CHN	2013
KY928861	*Marmota himalayana*	CHN	2013

KY928862	*Marmota himalayana*	CHN	2013
KY928863	*Marmota himalayana*	CHN	2013
KY928864	*Marmota himalayana*	CHN	2013
KY928865	*Marmota himalayana*	CHN	2013
KY928866	*Marmota himalayana*	CHN	2013
KY928867	*Marmota himalayana*	CHN	2013
KY928868	*Marmota himalayana*	CHN	2013
KY928869	*Marmota himalayana*	CHN	2013
KY928870	*Marmota himalayana*	CHN	2013
KY928871	*Marmota himalayana*	CHN	2013
KY928872	*Marmota himalayana*	CHN	2013
KY928873	*Marmota himalayana*	CHN	2013
KY928874	*Marmota himalayana*	CHN	2013
KY928875	*Marmota himalayana*	CHN	2013
KY928876	*Marmota himalayana*	CHN	2013
KY928877	*Marmota himalayana*	CHN	2013
KY928878	*Marmota himalayana*	CHN	2013
KY928879	*Marmota himalayana*	CHN	2013
KY928880	*Marmota himalayana*	CHN	2013
KY928881	*Marmota himalayana*	CHN	2013
KY928882	*Marmota himalayana*	CHN	2013
KY928883	*Marmota himalayana*	CHN	2013
KY928884	*Marmota himalayana*	CHN	2013
KY928885	*Marmota himalayana*	CHN	2013
KY928886	*Marmota himalayana*	CHN	2013
KY928887	*Marmota himalayana*	CHN	2013
KY928888	*Marmota himalayana*	CHN	2013
KY928889	*Marmota himalayana*	CHN	2013
KY928890	*Marmota himalayana*	CHN	2013
KY928891	*Marmota himalayana*	CHN	2013
KY928892	*Marmota himalayana*	CHN	2013
KY928893	*Marmota himalayana*	CHN	2013
KY928894	*Marmota himalayana*	CHN	2013
KY928895	*Marmota himalayana*	CHN	2013
KY928896	*Marmota himalayana*	CHN	2013
KY928897	*Marmota himalayana*	CHN	2013
KY928898	*Marmota himalayana*	CHN	2013
KY928899	*Marmota himalayana*	CHN	2013
KY928900	*Marmota himalayana*	CHN	2013

KY928901	*Marmota himalayana*	CHN	2013
KY928902	*Marmota himalayana*	CHN	2013
KY928903	*Marmota himalayana*	CHN	2013
KY928904	*Marmota himalayana*	CHN	2013
KY928905	*Marmota himalayana*	CHN	2013
KY928906	*Marmota himalayana*	CHN	2013
KY928907	*Marmota himalayana*	CHN	2013
KY928908	*Marmota himalayana*	CHN	2013
KY928909	*Marmota himalayana*	CHN	2013
KY928910	*Marmota himalayana*	CHN	2013
KY928911	*Marmota himalayana*	CHN	2013
KY928912	*Marmota himalayana*	CHN	2013
KY928913	*Marmota himalayana*	CHN	2013
KY928914	*Marmota himalayana*	CHN	2013
KY928915	*Marmota himalayana*	CHN	2013
KY928916	*Marmota himalayana*	CHN	2013
KY928917	*Marmota himalayana*	CHN	2013
KY928918	*Marmota himalayana*	CHN	2013
KY928919	*Marmota himalayana*	CHN	2013
KY928920	*Marmota himalayana*	CHN	2013
KY928921	*Marmota himalayana*	CHN	2013
KY928922	*Marmota himalayana*	CHN	2013
KY928923	*Marmota himalayana*	CHN	2013
KY928924	*Marmota himalayana*	CHN	2013
KY928925	*Marmota himalayana*	CHN	2013
KY928926	*Marmota himalayana*	CHN	2013
KY928927	*Marmota himalayana*	CHN	2013
KY928928	*Marmota himalayana*	CHN	2013
KY928929	*Marmota himalayana*	CHN	2013
KY928930	*Marmota himalayana*	CHN	2013
KY928931	*Marmota himalayana*	CHN	2013
KY928932	*Marmota himalayana*	CHN	2013
KY928933	*Marmota himalayana*	CHN	2013
KY928934	*Marmota himalayana*	CHN	2013
KY928935	*Marmota himalayana*	CHN	2013
KY928936	*Marmota himalayana*	CHN	2013
KY928937	*Marmota himalayana*	CHN	2013
KY928938	*Marmota himalayana*	CHN	2013
KY928939	*Marmota himalayana*	CHN	2013

KY928940	*Marmota himalayana*	CHN	2013
KY928941	*Marmota himalayana*	CHN	2013
KY928942	*Marmota himalayana*	CHN	2013
KY928943	*Marmota himalayana*	CHN	2013
KY928944	*Marmota himalayana*	CHN	2013
KY928945	*Marmota himalayana*	CHN	2013
KY928946	*Marmota himalayana*	CHN	2013
KY928947	*Marmota himalayana*	CHN	2013
KY928948	*Marmota himalayana*	CHN	2013
KY928949	*Marmota himalayana*	CHN	2013
KY928950	*Marmota himalayana*	CHN	2013
KY928951	*Marmota himalayana*	CHN	2013
KY928952	*Marmota himalayana*	CHN	2013
KY928953	*Marmota himalayana*	CHN	2013
KY928954	*Marmota himalayana*	CHN	2013
KY928955	*Marmota himalayana*	CHN	2013
KY928956	*Marmota himalayana*	CHN	2013
KY928957	*Marmota himalayana*	CHN	2013
KY928958	*Marmota himalayana*	CHN	2013
KY928959	*Marmota himalayana*	CHN	2013
KY928960	*Marmota himalayana*	CHN	2013
KY928961	*Marmota himalayana*	CHN	2013
KY928962	*Marmota himalayana*	CHN	2013
KY928963	*Marmota himalayana*	CHN	2013
KY928964	*Marmota himalayana*	CHN	2013
KY928965	*Marmota himalayana*	CHN	2013
KY928966	*Marmota himalayana*	CHN	2013
KY928967	*Marmota himalayana*	CHN	2013
KY928968	*Marmota himalayana*	CHN	2013
KY928969	*Marmota himalayana*	CHN	2013
KY928970	*Marmota himalayana*	CHN	2013
KY928971	*Marmota himalayana*	CHN	2013
KY928972	*Marmota himalayana*	CHN	2013
KY928973	*Marmota himalayana*	CHN	2013
KY928974	*Marmota himalayana*	CHN	2013
KY928975	*Marmota himalayana*	CHN	2013
KY928976	*Marmota himalayana*	CHN	2013
KY928977	*Marmota himalayana*	CHN	2013
KY928978	*Marmota himalayana*	CHN	2013

KY928979	*Marmota himalayana*	CHN	2013
KY928980	*Marmota himalayana*	CHN	2013
KY928981	*Marmota himalayana*	CHN	2013
KY928982	*Marmota himalayana*	CHN	2013
KY928983	*Marmota himalayana*	CHN	2013
KY928984	*Marmota himalayana*	CHN	2013
KY928985	*Marmota himalayana*	CHN	2013
KY928986	*Marmota himalayana*	CHN	2013
KY928987	*Marmota himalayana*	CHN	2013
KY928988	*Marmota himalayana*	CHN	2013
KY928989	*Marmota himalayana*	CHN	2013
KY928991	*Marmota himalayana*	CHN	2013
KY928992	*Marmota himalayana*	CHN	2013
KY928993	*Marmota himalayana*	CHN	2013
KY928994	*Marmota himalayana*	CHN	2013
KY928995	*Marmota himalayana*	CHN	2013
KY928996	*Marmota himalayana*	CHN	2013
KY928997	*Marmota himalayana*	CHN	2013
KY928998	*Marmota himalayana*	CHN	2013
KY928999	*Marmota himalayana*	CHN	2013
KY929000	*Marmota himalayana*	CHN	2013
KY929001	*Marmota himalayana*	CHN	2013
KY929002	*Marmota himalayana*	CHN	2013
KY929003	*Marmota himalayana*	CHN	2013
KY929005	*Marmota himalayana*	CHN	2013
KY929006	*Marmota himalayana*	CHN	2013
KY929007	*Marmota himalayana*	CHN	2013
KY929008	*Marmota himalayana*	CHN	2013
KY929009	*Marmota himalayana*	CHN	2013
KY929010	*Marmota himalayana*	CHN	2013
KY929011	*Marmota himalayana*	CHN	2013
KY929012	*Marmota himalayana*	CHN	2013
LC110352	*Mus musculus*	JPN	2015
LC337994	*Camelus dromedaries*	ARE	2013
LC337995	*Camelus dromedaries*	ARE	2013
LC337996	*Camelus dromedaries*	ARE	2013
LC337997	*Camelus dromedaries*	ARE	2013
LC337998	*Camelus dromedaries*	ARE	2013
LC338000	*Camelus dromedaries*	ARE	2013

MG010885	*Macaca mulatta*	TCD	2011
MG010886	*Macaca mulatta*	TCD	2011
MG010887	*Macaca mulatta*	TCD	2011
MG010888	*Macaca mulatta*	TCD	2011
MG010889	*Macaca mulatta*	TCD	2011
MG010890	*Macaca mulatta*	TCD	2011
MG010891	*Macaca mulatta*	TCD	2011
MG010892	*Macaca mulatta*	TCD	2011
MG010893	*Macaca mulatta*	TCD	2011
MG010894	*Macaca mulatta*	TCD	2011
MG010895	*Macaca mulatta*	TCD	2011
MG010896	*Macaca mulatta*	TCD	2011
MG010898	*Macaca mulatta*	TCD	2011
MG190028	*Roe deer*	SVN	2014
MG600063	*Parupeneus cyclostomus*	CHN	NA
MG846392	*Gallus gallus*	BRA	2015
MG846393	*Gallus gallus*	BRA	2015
MG846395	*Gallus gallus*	BRA	2015
MG846396	*Gallus gallus*	BRA	2015
MG846397	*Gallus gallus*	BRA	2015
MG846398	*Gallus gallus*	BRA	2015
MG846399	*Gallus gallus*	BRA	2015
MG846400	*Gallus gallus*	BRA	2015
MH327933	*Gallus gallus*	HUN	2011
MH425579	*Gallus gallus*	HUN	2013
MH425580	*Gallus gallus*	HUN	2013
MH425581	*Gallus gallus*	HUN	2013
MH425582	*Gallus gallus*	HUN	2013
MH425583	*Gallus gallus*	HUN	2013
MH453876	*Australian Shelduck*	AUS	2012
MH453877	*Australian Shelduck*	AUS	2012
MK204399	*Anas gracilis*	AUS	2017
MK204419	*Malacorhynchus membranaceus*	AUS	2017
MK305309	*Homo sapiens*	AGO	2018
MK378829	*Sus scrofa*	CHN	2004
MK378842	*Sus scrofa*	CHN	2004
MK378864	*Sus scrofa*	CHN	2004
MN692670	*Macaca fascicularis*	THA	2017
MT846991	*Pantholops hodgsonii*	CHN	2014

MT846992	*Pantholops hodgsonii*	CHN	2014
MT846993	*Pantholops hodgsonii*	CHN	2014
MT846994	*Pantholops hodgsonii*	CHN	2014
MT846995	*Pantholops hodgsonii*	CHN	2014
MT846996	*Pantholops hodgsonii*	CHN	2014
MT846997	*Pantholops hodgsonii*	CHN	2014
MT846998	*Pantholops hodgsonii*	CHN	2014
MT846999	*Pantholops hodgsonii*	CHN	2014
NC-029801	*Sus scrofa*	ITA	2004
NC-034160	*Zalophus californianus*	AGO	2008
NC-040438	*Gallus gallus*	HUN	2011
NC-040752	*Roe deer*	SVN	2014
KC692367	*Vulpes vulpes*	NLD	2012
KY502844	*Gorilla gorilla*	COD	2015
AB186897	*NA*	THA	NA

Supplemental Table S2. Capsid sequences of PBV used in the current study for phylogenetic analysis.

Sample	Virus	Virus reads	Bacteria
1-sputum-4279	-	-	*Burkholderia*
2-sputum-3406	porcine picobirnavirus-3	2	*Haemophilus*
3-sputum-4940	influenza A	8	-
4-sputum-4942	-	-	-
5-sputum-4242	-	-	*Klebsiella, Stenotrophomonas*
6-sputum-4246	-	-	-
7-sputum-4256	-	-	*Acinetobacter, Klebsiella*
8-sputum-4366	HHV-4	79	-
9-sputum-4352	Aichivirus, HHV-4, HHV-7	65, 42, 61	-
10-sputum-4946	-	-	-
11-sputum-4938	HHV-1	6045	*Stenotrophomonas*
12-sputum-4947	HHV-1	30	-
13-sputum-4395	-	-	-
14-sputum-4418	-	-	-
15-sputum-4419	-	-	-
Ctrl-sputum-3405	HHV-4	30	*Acinetobacter, Neisseria, Klebsiella*

Supplemental Table S3. Viral and bacterial infections detected in primary screen. 16 sputum samples from individuals in Colombia were sequenced by mNGS. Viral species (read numbers listed) and bacterial genera enriched are listed. (-): nothing detected.

Analysis	Statistic	IR(CI95%)	Observed mean (CI 95%)	Expected mean (CI95%)	Significance
**RdRp**	AI	0.14 (0.11-0.19)	0.27 (0.25-0.28)	1.90 (1.32-2.44)	<0.0001
	PS	0.22 (0.22-0.25)	2.00 (2.00-2.00)	8.92 (8.04-9.00)	<0.0001
	MC (Non-CRC)	ND	216.50 (213.0-219.0)	71.78 (36.00-103.0)	<0.001
	MC (CRC)	ND	4.20 (4.00-6.00)	1.05 (1.00-1.35)	<0.001
**Capsid**	AI	$1.9x10^{-4}$ ($0.3x10^{-7}$-0.003)	0.001 ($1.3x10^{-7}$-0.002)	5.3 (4.39-6.16)	<0.0001
	PS	0.08(0.09-0.08)	2 (2.0-2.0)	24.48 (23.17-25.0)	<0.0001
	MC (Non-CRC)	ND	112.98 (78.0-126.0)	36.19 (23.46-58.0)	0.001
	MC (CRC)	ND	19.0 (19.0-19.0)	1.38 (1.0-2.0)	<0.0001

Supplemental Table S4. Phylogeny-trait association tests of the clinical condition structure (CRC) of PBV RdRp and capsid coding sequences using BaTS.
